# Human Dendritic Cell Subsets Undergo Distinct Metabolic Reprogramming for Immune Response

**DOI:** 10.3389/fimmu.2018.02489

**Published:** 2018-11-01

**Authors:** Farhan Basit, Till Mathan, David Sancho, I. Jolanda M. de Vries

**Affiliations:** ^1^Department of Tumor Immunology, Radboud Institute for Molecular Life Sciences, Radboud University Medical Center, Nijmegen, Netherlands; ^2^Centro Nacional de Investigaciones Cardiovasculares Carlos III, Madrid, Spain; ^3^Department of Medical Oncology, Radboud University Medical Center, Nijmegen, Netherlands

**Keywords:** CD1c^+^ mDC, pDC, glutaminolysis, mitophagy, mitochondrial dynamics, OXPHOS, glycolysis

## Abstract

Toll-like receptor (TLR) agonists induce metabolic reprogramming, which is required for immune activation. We have investigated mechanisms that regulate metabolic adaptation upon TLR-stimulation in human blood DC subsets, CD1c^+^ myeloid DCs (mDCs) and plasmacytoid DCs (pDCs). We show that TLR-stimulation changes expression of genes regulating oxidative phosphorylation (OXPHOS) and glutamine metabolism in pDC. TLR-stimulation increases mitochondrial content and intracellular glutamine in an autophagy-dependent manner in pDC. TLR-induced glutaminolysis fuels OXPHOS in pDCs. Notably, inhibition of glutaminolysis and OXPHOS prevents pDC activation. Conversely, TLR-stimulation reduces mitochondrial content, OXPHOS activity and induces glycolysis in CD1c^+^ mDC. Inhibition of mitochondrial fragmentation or promotion of mitochondrial fusion impairs TLR-stimulation induced glycolysis and activation of CD1c^+^ mDCs. TLR-stimulation triggers BNIP3-dependent mitophagy, which regulates transcriptional activity of *AMPK*α*1*. BNIP3-dependent mitophagy is required for induction of glycolysis and activation of CD1c^+^ mDCs. Our findings reveal that TLR stimulation differentially regulates mitochondrial dynamics in distinct human DC subsets, which contributes to their activation.

## Introduction

Dendritic cells (DCs) regulate the immune homeostasis and development of adaptive immune responses. In human peripheral blood, there are two main subsets of naturally circulating DCs, namely CD1c^+^ myeloid dendritic cells (CD1c^+^ mDCs) and plasmacytoid dendritic cells (pDC) (1, 2). These subsets differ in function, localization, and phenotype. CD1c^+^ mDCs are primarily localized in the marginal zone of the lymph nodes and confer immunity against bacteria and fungi (3, 4) by inducing Th1 responses *via* the production of IL-12 (5, 6). Conversely, pDCs localize to the T-cell areas in lymph nodes and are proficient in viral antigen recognition (7). Mature pDCs abundantly produce type I IFNs upon activation and induce T cell responses (2, 8).

Under non-inflammatory conditions, DCs are poorly immunogenic. However, inflammatory stimuli or pathogen-derived products trigger a group of pattern recognition receptors, including Toll-like receptors (TLRs), which results in a process of cellular activation, termed DC maturation, hence making them highly immunogenic (9). DC maturation is a tightly coordinated response, which involves various signaling pathways, molecular trafficking, cytokine production and cytoskeletal remodeling (10–12). These processes require metabolic adaptations, which are essential for DC survival, migration and eventually the development of immunity. DC activation upon TLR stimulation is associated with metabolic reprogramming and expression of genes encoding cytokines and chemokines, which promote immune response (13, 14). Effector functions requires a glycolytic switch in mouse bone-marrow DCs cultured in GM-CSF (14, 15), while lipid metabolism and OXPHOS are indispensable for murine pDC immune function (16).

Mitochondrial dynamics and bioenergetics are reciprocally coupled to adjust bioenergetic adaptation to metabolic needs of the cell (17). Mitochondrial dynamics are controlled by a group of dynamin-related GTPases, i.e., mitofusin 1 and 2 (Mfn1/2) and optic atrophy 1 (Opa1) for fusion and dynamin related protein 1 (Drp1) for fission (18). Mfn1 plays a crucial role in mitochondrial fusion, while Mfn2 is central to mitochondrial metabolism, by regulating mitochondrial membrane potential and the OXPHOS system (17). The balanced mitochondrial dynamics is critical for normal mitochondrial function, bioenergetics and quality control *via* mitophagy (19–21). Mitophagy is a process by which a cell removes damaged mitochondria to use them as additional fuels during stress (22, 23). Upon stress or damage, mitochondria exhibit compromised metabolism, ATP production and reduction in membrane potential, which are characteristics of mitochondrial dysfunction and the initial trigger for mitophagy (24).

Understanding of metabolic changes underpinning human DC-subsets immune function are less known and insights into these changes can help develop new strategies for controlling immunogenicity. Given the distinct ontogeny and functional specializations of CD1c^+^ mDC and pDC, we aimed at identifying metabolic adaptations engaged by human DC-subsets for effector function. We here demonstrate that TLR-stimulation in CD1c^+^ mDC and pDC results in differential mitochondrial rewiring and metabolic adaptations. TLR stimulation results in increased glutaminolysis and OXPHOS in pDC, while it promotes mitophagy and glycolysis in CD1c^+^ mDC. Notably, these metabolic adaptations are indispensable for activation of CD1c^+^ mDC and pDC. Our data provides novel insights into subset-specific regulation of mitochondrial metabolism, which impacts DC function.

## Materials and methods

### Chemicals

Mdivi-1 (#M0199), Niclosamide (#N3510), 6-Diazo-5-oxo-L-norleucine (#D2141), 2-Deoxy-D-glucose (#D8375), BPTES (#SML0601), Chloroquine (#C6628), 3-Methyladenine (#M9281), Poly-D-lysine hydrobromide (#P7280), Antimycin A (#A8674), Oligomycin A (#O4876) and Rotenone (#R8875) were obtained from Sigma-Aldrich. Olomoucine (#10010240) was obtained from Caymanchem. Piericidin A (#ALX-380-235-M002) was obtained from Enzo Life Sciences. MitoTracker™ Green FM (#M7514), MitoTracker™ Red CMXRos (#M7512) and 2-NBDG (#N13195) were obtained from Thermo Fisher Scientific. EnzyChrom™ Glutamine Assay Kit (#EGLN-100) was purchased from BioAssay Systems. 15-oxospiramilactone (S3) was kindly provided by Prof. Xiaojiang Hao (The State Key Laboratory of Phytochemistry and Plant Resources in West China, Kunming Institute of Botany, Chinese Academy of Sciences, Kunming, Yunnan 650204, China). SF2312 was kindly provided by Dr. Florian Muller (The University of Texas MD Anderson Cancer Center, USA).

Cytokine detection–Supernatant was taken from each sample after overnight incubation and analyzed with standard sandwich ELISAs to detect TNF-α using human TNF-α ELISA Kit (#88-7346-22) from Thermo Fisher Scientific and IFN-α (#BMS216INSTCE) from Bender Medsystems, Vienna.

### DC isolation and culture

For functional assays, DCs were isolated from buffy coats of healthy volunteers (Sanquin, Nijmegen, The Netherlands). Written informed consent per the Declaration of Helsinki and according to institutional guidelines, were obtained from healthy volunteers. Peripheral blood mononuclear cells (PBMCs) were isolated by using Ficoll density centrifugation (Lymphoprep; Axis-Shield PoC AS, Oslo, Norway). CD1c isolation kit (Miltenyi Biotec, Bergisch-Gladbach, Germany) was used to isolate CD1c^+^ mDCs, as per manufacturer's instructions. Next, monocytes were depleted by either plastic adhesion, or by the use of CD14 microbeads (Miltenyi Biotec). Consequently, pDCs were purified by positive selection using anti–BDCA-4–conjugated magnetic microbeads (Miltenyi Biotec). DCs were cultured in X-VIVO-15 medium (Lonza, Basel, Switzerland) supplemented with 2% human serum (Sanquin). DCs were stimulated with: pRNA (15 μg/ml) freshly prepared 5–10 min before adding to the cell culture. pDCs were cultured with IL-3 (10 ng/mL) (Cellgenix, Freiburg, Germany) as a survival factor in addition to the stimuli.

### Flow cytometry

The phenotype of pDC and CD1c^+^ mDC populations was determined by flow cytometry. DC purity was assessed by double staining CD11c^+^/CD1c^+^ for CD1c^+^ mDCs (above 95%) and BDCA2/CD123 for pDCs (above 95%; all Miltenyi Biotec) (25). The following primary monoclonal antibodies (mAbs) were used to determine the maturation state of the DCs: anti–CD80-APC, anti–PD-L1-APC (all BD Bioscience, San Jose, CA). Anti-BNIP-3 Antibody (#sc-56167 FITC) was purchased from Santa Cruz Biotechnology. Anti-Mfn2 (#M6444) and Anti-Drp1 (#ABT155) were purchased from Sigma-Aldrich Anti-Porin (#529536) was purchased from Calbiochem. Anti-NDUFA10 (#ab174829) was purchased from abcam. Autophagosomes were detected using Autophagy detection kit (Enzo Life Sciences # ENZ 51031-0500) according to the manufacturer's instructions. Briefly, cells were incubated with CYTO-ID Green autophagy detection dye (1:2,000) for 30 min at 37°C. Subsequently, cells were washed and analyzed by flow cytometry. Cell viability was determined using Fixable Viability Dye eFluor™ 780 (Invitrogen # 65-0865-14) according to manufacturer's instructions. Briefly, cells were incubated with Fixable Viability Dye eFluor™ 780 (1:2000) at 4°C for 20 min. Subsequently, cells were washed and analyzed by flow cytometry. Measurements were performed on FACSVerse flowcytometers (BD).

### Metabolism assay

An XF-96 Extracellular Flux Analyzer (Seahorse Bioscience) was used for Extracellular flux analyses of CD1c^+^ mDC and pDCs (50,000 cells/well) (26). For mitochondrial fitness tests, OCR was measured sequentially at basal, and following the addition of 1 μM oligomycin, 3 μM FCCP (fluorocarbonyl cyanide phenylhydrazone), 1 μM ROT + 1 μM AA. Intracellular concentrations of glutamine were determined using a quantitative colorimetric enzyme assay kit (#EGLN-100; BioAssay Systems, Hayward, CA). Samples were diluted (1:2) with distilled water. All materials and chemicals were provided by the manufacturer, and manufacturer's instruction were followed.

### Protamine-RNA complexes

pRNA complexes were made freshly before adding to the cells. Protamine (protaminehydrochloride MPH 5000 IE/ml; Meda Pharma BV Amstelveen, The Netherlands) was diluted to 0.5 mg/ml in RNase free water and mixed with 2-kbp-long single-stranded mRNA (coding for gp100). It was extensively mixed and incubated for 5–10 min at room temperature, before added to the cells.

### Quantitative real-time PCR (qPCR)

qPCR was carried out in 25-μl reaction mixture containing 2 μl of cDNA, 12.5 μl of SYBR Green master mix (Applied Biosystems #A25742, Austin, USA) and 250 nmol of forward and reverse primer. The reaction conditions were as follows: 50°C for 2 min, 95°C for 10 min and then 40 cycles of 95°C for 15 s and 60°C for 1 min. For qPCR following primer sequences were used; AMPK1α forward, 5′-TGCGTGTACGAAGGAAGAATCC-3′ and reverse, 5′-TGTGACTTCCAGGTCTTGGAGTT-3′; β-Actin forward, 5′-TGACAGGATCGAGAAGGAGA-3′ and reverse 5′-CGCTCAGGAGGAGCAATG-3′.

### RNA sequencing

Total RNA was isolated from CD1c^+^ mDCs and pDCs using Trizol (Invitrogen, MA, USA). RNA sequencing and read alignment were performed by BGI TECH SOLUTIONS (Hong Kong). Reads were aligned to human genome version 19. RNA sequencing data is deposited at the Gene Expression Omnibus (GEO; accession number: GSE89442). Data was analyzed using the R platform package “edgeR,” version 3.12, to analyze whole transcriptome principal coordinates analysis (using the “plotMDS” command), differential expression analysis, and GO term analysis. Differential expression was determined by fitting a generalized linear model using the “glmFit” command, and significance was determined using the likelihood ratio test provided by the “glmLRT” command (27).

## Results

### Mitochondrial dynamics is differentially regulated in CD1c^+^ mDC and pDC upon TLR7/8 stimulation

**Figure 1:**
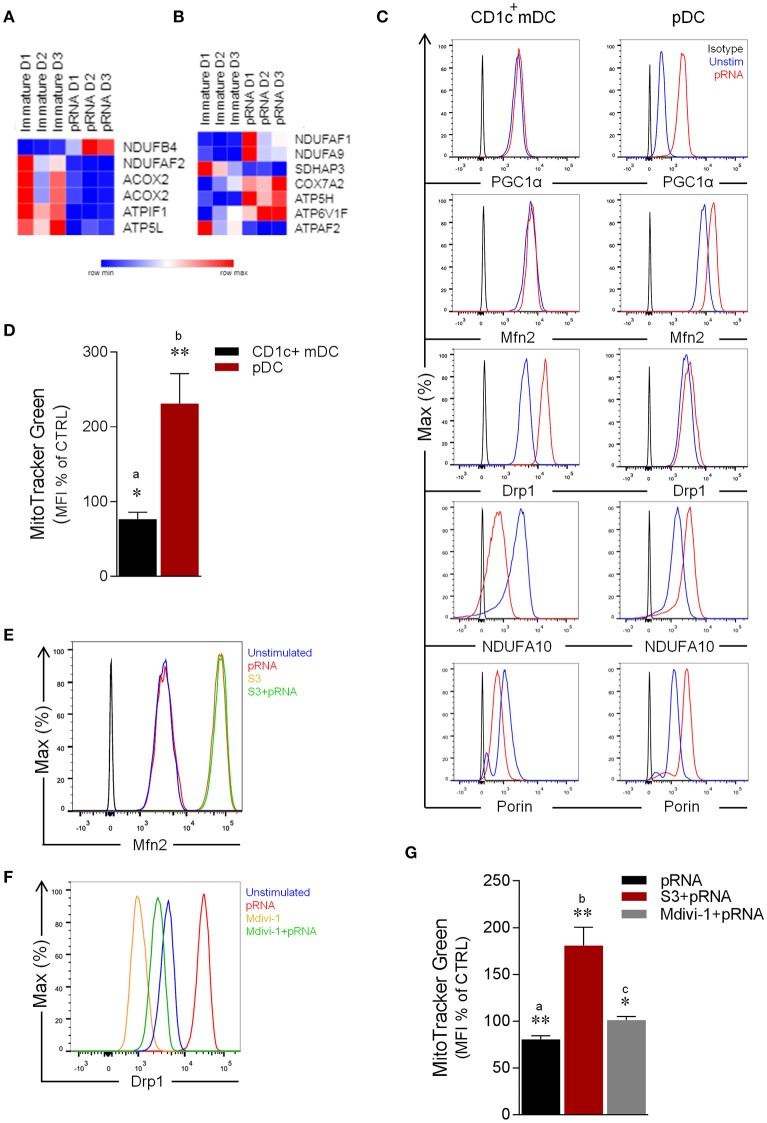
Effect of pRNA on mitochondrial dynamics in CD1c^+^ mDC and pDC. **(A)** Heatmap showing expression of significantly changed genes which regulate OXPHOS in CD1c^+^ mDC upon pRNA-stimulation. Red color indicates increased expression while blue color shows decreased expression. **(B)** Heatmap showing expression of significantly changed genes which regulate OXPHOS in pDC upon pRNA-stimulation. Red color indicates increased expression while blue color shows decreased expression. **(C)** Flow cytometry histograms of PGC1α, Mfn2, NDUFA10, Porin and Drp1 in Drp1 in CD1c^+^ mDC and pDC. Black represents isotype control, blue represents unstimulated control and red represents pRNA stimulated cells for 6 h. **(D)** Percentage mean fluorescence intensity of cells stained with MitoTracker Green FM and stimulated with pRNA for 6 h. Data represents mean ± SEM of four independent experiments ^*^p < 0.05; ^**^p < 0.01 (Student's *t*-test). **(E)** Flow cytometry histograms of Mfn2 in CD1c^+^ mDC. Blue represents unstimulated control, red represents pRNA stimulated cells for 6 h, brown represents S3 and green represents S3+pRNA. **(F)** Flow cytometry histograms of Drp1 in CD1c^+^ mDC. Blue represents unstimulated control, red represents pRNA stimulated cells, brown represents Mdivi-1 and green represents Mdivi-1+pRNA. **(G)** Percentage mean fluorescence intensity of cells stained with MitoTracker Green FM and stimulated with pRNA for 6 h in the presence or absence of 5 μM S3 or 1 μM Mdivi-1. Data represents mean ± SEM of four independent experiments. ^*^p < 0.05; ^**^p < 0.01 (Student's *t*-test).

To investigate changes in metabolism, human CD1c^+^ mDC and pDC were stimulated with a complex of protamine and mRNA (pRNA) that acts as a TLR7/8 ligand. pRNA has been shown to activate CD1c^+^ mDCs and pDCs and induces them to release IL-12 and IFN-α, respectively (28). Previously, we analyzed the whole-transcriptome of human CD1c^+^ mDC and pDC upon TLR7/8 stimulation (27). Our data demonstrated that pRNA upregulated cytokines and migration-related genes in CD1c^+^ mDCs as well as type I and III interferons (IFN-α and IFN-λ) related genes in pDC. Moreover, we demonstrated that pRNA stimulation increased expression of maturation markers (i.e., CD80, PD-L1 & CD40) in both CD1c^+^ mDC and pDC, in addition to increase in immunostimulatory cytokines i.e., TNFα and INFα for CD1c^+^ mDC and pDC, respectively (27). To investigate whether changes in metabolism are required for human DC-subsets immune response, we analyzed expression of OXPHOS related genes in human CD1c^+^ mDC and pDC. OXPHOS related genes were significantly downregulated in CD1c^+^ mDCs upon pRNA-stimulation (Figure 1A). Conversely pRNA-stimulation increased expression of *NDUFAF1, NDUFA9, COX7A2, ATP5H*, and *ATP6V1F* in pDC (Figure 1B) suggesting up-regulation of OXPHOS in pDC.

To explore the question whether TLR-stimulation modulates OXPHOS, we next examined the effect of pRNA on NDUFA10 protein, which is an accessory subunit of the mitochondrial respiratory chain complex I (29). Importantly, pRNA stimulation reduced NDUFA10 in CD1c^+^ mDC, in comparison to increase of NDUFA10 in pDC (Figure 1C). Given, the crucial role of Mfn2 and Drp1 in regulating OXPHOS system and metabolism (17, 30–32), we analyzed the effect of TLR-stimulation on Mfn2 and Drp1 protein levels. Intriguingly, analysis of protein expression revealed that pRNA-stimulation increased levels of Drp1 in CD1c^+^ mDC whereas Mfn2 levels remained unchanged (Figure 1C). Conversely, in pDC, pRNA-stimulation increased Mfn2 protein levels, whereas Drp1 protein levels remained unchanged (Figure 1C). Peroxisome proliferator-activated receptor gamma coactivator 1-alpha (PGC-1α) controls mitochondrial biogenesis, oxidative phosphorylation (33, 34) and mitochondrial dynamics (35, 36). TLR7/8-stimulation increased PGC-1α expression in pDC, whereas it had no effect on PGC-1α expression in CD1c^+^ mDC (Figure 1C). The Voltage-Dependent Anion Channel (VDAC or porin) is an outer membrane mitochondrial protein, which is implicated in alteration of mitochondrial morphology (37). Importantly, pRNA-stimulation reduced porin levels in CD1c^+^ mDC and increased porin levels in pDC (Figure 1C). Of note, pRNA-stimulation did not affect viability of CD1c^+^ mDC and pDC (Supplementary Figures 4, 6).

Based on these findings, we hypothesized that TLR7/8-stimulation alters mitochondrial content in CD1c^+^ mDC. To test this, CD1c^+^ mDC were stained with MitoTracker™ Green FM, a fluorescent dye that localizes to mitochondria in a mitochondrial membrane potential independent manner. Indeed, TLR7/8-stimulation significantly decreased mitochondrial content in CD1c^+^ mDC (Figure 1D). By comparison, staining of pDC with MitoTracker™ Green FM showed a significant increase in mitochondrial content upon TLR7/8-stimulation (Figure 1D) consistent with increased Mfn2 and PGC1α levels. To confirm the involvement of mitochondrial dynamics in regulating mitochondrial mass, we stimulated CD1c^+^ mDC with pRNA in the presence or absence of a fusion promoter (15-oxospiramilactone, S3) (38) or a fission inhibitor (Mdivi-1) (39). Interestingly, S3 increased Mfn2 expression and Mdivi-1 reduced both endogenous and pRNA-induced Drp1 levels in CD1c^+^ mDC (Figures 1E,F). Of note, S3 and Mdivi-1 significantly prevented loss of mitochondrial content in CD1c^+^ mDC upon TLR7/8-stimulation (Figure 1G). Collectively, these data indicate that TLR7/8-stimulation results in mitochondrial fragmentation and reduced mitochondrial content in CD1c^+^ mDCs and increased mitochondrial biogenesis, fusion and content in pDCs.

### pDC stimulated via TLR7/8 have increased glutaminolysis and OXPHOS which are crucial for activation

**Figure 2:**
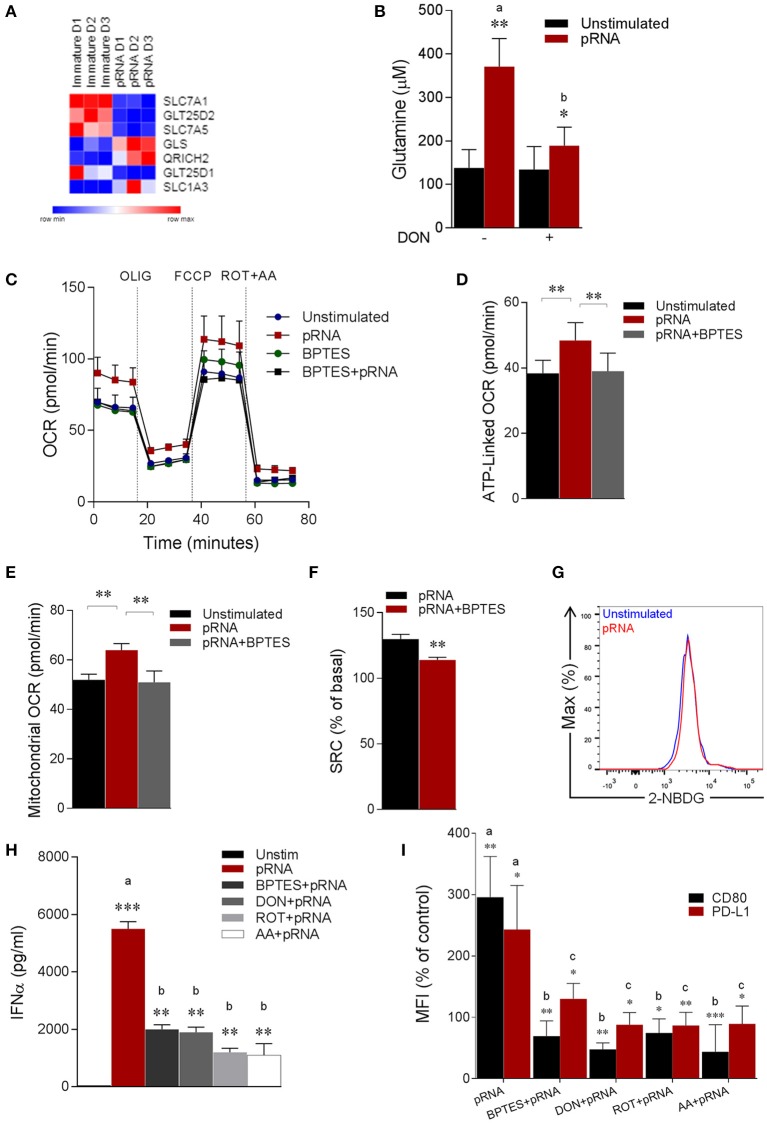
pDC stimulated with pRNA have increased glutaminolysis and OXPHOS which are required for activation. **(A)** Heatmap showing expression of significantly changed genes which regulate amino acid metabolism in pDCs upon pRNA-stimulation for 6 h. Red color indicates increased expression while blue color shows decreased expression. **(B)** Glutamine concentration measured by a coupled glutaminase, glutamate dehydrogenase assay with correction for glutamate concentration. Data represents mean ± SEM of experiments from six donors. ^*^p < 0.05; ^**^p < 0.01 (Student's *t*-test). **(C)** Mitochondrial fitness test of pDCs stimulated with pRNA for 6 h in the presence or absence of 5 μM BPTES. Data represents mean ± SEM of three independent experiments. **(D–F)** Data was collected within same experiments as C, but is shown separately for better understanding. Data represents mean ± SEM of three independent experiments. ^*^p < 0.05; ^**^p < 0.01 (Student's *t*-test). **(G)** Flow cytometry histograms of 2-NBDG stained pDCs. Blue represents unstimulated control and red represents pRNA-stimulated cells pDC for 6 h. **(H)** IFN-α levels on protein level were measured in the supernatant of the pDCs stimulated for 6 h. Data represents mean ± SEM of three independent experiments ^**^p < 0.01; ^***^p < 0.001 (Student's *t*-test). **(I)** Percentage mean flouresence intensity of maturation markers (CD80 and PD-L1) in pDCs stimulated for 6 h. Data represents mean ± SEM of three independent experiments. ^*^p < 0.05; ^**^p < 0.01; ^***^p < 0.001 (Student's *t*-test).

We next asked whether increased mitochondrial fusion and content along with upregulation of NDUFA10 and OXPHOS related genes in TLR7/8-stimulated pDCs was associated with metabolic changes. OXPHOS is driven by NADH and FADH_2_, produced by the tricarboxylic acid (TCA) cycle (40, 41) and the amino acid glutamine is among the key metabolites that support the TCA cycle. Glutaminolysis is a metabolic pathway, which requires deamination of glutamine by glutaminase (GLS), generating glutamate, which in turn is converted to α-KG, a TCA cycle intermediate (42, 43). To determine whether glutaminolysis contributes to increased OXPHOS upon TLR7/8-stimulation in pDC, we examined expression of genes related to amino acid metabolism. pRNA-stimulation significantly increased expression of *GLS* and *SLC1A3* in pDC (Figure 2A). *GLS* catalyzes the conversion of glutamine to glutamate (44) while *SLC1A3* is a glutamate transporter (45, 46). Upregulation of these genes suggests increased glutaminolysis in pDCs upon TLR-stimulation. To test this, we measured intracellular glutamine levels in pDC. pRNA-stimulation significantly increased intracellular glutamine in pDC, which could be inhibited by 6-Diazo-5-oxo-L-norleucine (DON) (Figure 2B), a glutamine antagonist, which inhibits glutamine utilizing enzymes by irreversible alkylation of L-cysteinyl residues (47).

Notably, extracellular flux analysis (EFA) revealed increased basal oxygen consumption rate (OCR), maximal OCR (Figure 2C; Supplementary Figures 1B,C), ATP-linked OCR, mitochondrial OCR and spare respiratory capacity (SRC) in pRNA-stimulated pDC compared to unstimulated pDC (Figures 2D–F). To explore whether increased OXPHOS activity in pRNA-stimulated pDC is due to increased glutaminolysis, we pharmacologically attenuated Glutaminase, an enzyme responsible for conversion of glutamine into glutamate. pDC were stimulated with pRNA in the presence or absence of BPTES, a chemical inhibitor of GLS. BPTES inhibited in pDC the pRNA-induced increase in basal OCR (Figure 2C; Supplementary Figures 1A–C), ATP-linked OCR (Figure 2D), maximal OCR (Supplementary Figure 1C) mitochondrial OCR (Figure 2E) and SRC (Figure 2F). These results indicate that pRNA stimulation of pDC results in increased OXPHOS due to increased glutaminolysis. Intriguingly, we did not observe an increase in ECAR (Supplementary Figure 1D) and 2-NBDG uptake (Figure 2G) upon pRNA-stimulation.

We next asked whether these metabolic changes are required for pDC activation. Activation of these cells was assessed by measuring secretion of immunostimulatory cytokine IFNα and membrane expression of co-stimulatory molecule CD80 and co-inhibitory molecule PD-L1. A reduced secretion of IFNα by pRNA-stimulated in pDC was observed when Rotenone (ROT), Antimycin A (AA), BPTES and DON were added to the culture medium (Figure 2H). Addition of these factors also significantly reduced the pRNA-mediated upregulation of CD80 and PD-L1 on pDC (Figure 2I). By comparison, we observed no effect of ROT, AA, BPTES and DON on pRNA-stimulated TNFα (Supplementary Figure 3B) and CD80 and PD-L1 in CD1c^+^ mDC (Supplementary Figure 3C).

**Figure 3:**
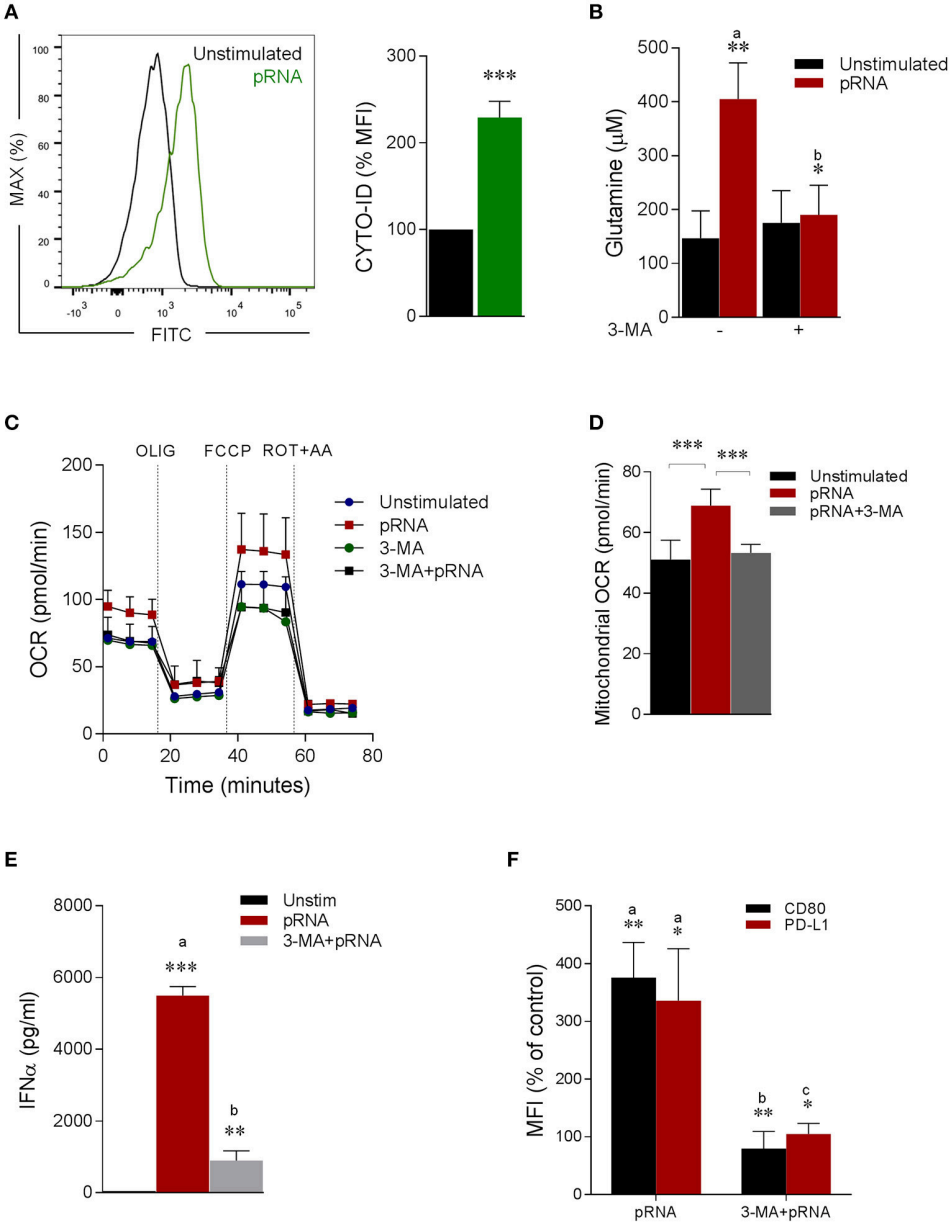
Autophagy provides glutamine for pDC activation. **(A)** Fluorescence intensity of autophagosomal marker CYTO-ID in pDC stimulated with pRNA for 6 h ^***^p < 0.001 (Student's *t*-test). **(B)** Glutamine concentration measured by a coupled glutaminase, glutamate dehydrogenase assay with correction for glutamate concentration. Data represents mean ± SEM of experiments from six donors. ^*^p < 0.05; ^**^p < 0.01 (Student's *t*-test). **(C)** Mitochondrial fitness test of pDCs stimulated with pRNA for 6 h in the presence or absence of 25 μM 3-MA. Data represents mean ± SEM of three independent experiments. **(D)** Data was collected within same experiments as **(C)** but is shown separately for better understanding. Data represents mean ± SEM of three independent experiments. ^**^p < 0.01; ^***^p < 0.001 (Student's *t*-test). **(E)** IFN-α levels on protein level were measured in the supernatant of the pDCs stimulated for 6 h. Data represents mean ± SEM of three independent experiments. ^**^p < 0.01; ^***^p < 0.001 (Student's *t*-test). **(F)** Percentage mean flouresence intensity of maturation markers (CD80 and PD-L1) in pDCs stimulated for 6 h. Data represents mean ± SEM of three independent experiments. ^*^p < 0.05; ^**^p < 0.01 (Student's *t*-test).

Of note, TLR stimulation triggers autophagy in pDC, which is required to produce type I IFN (48–52). Consistently, we observed significant increase in autophagosomes upon pRNA-stimulation in pDC (Figure 3A). Intriguingly, autophagy has been reported to supply metabolic substrates to preserve mitochondrial function (53–57). We hypothesized that increased glutamine and glutaminolysis in TLR7/8-stimulated pDCs is provided by autophagy. To investigate this, autophagy inhibitor 3-MA was added during the pRNA stimulation of pDC. 3-MA significantly reduced the pRNA-induced increase in glutamine levels in pDC (Figure 3B). Consistently, 3-MA significantly reduced pRNA-induced increase in basal OCR (Figure 3C; Supplementary Figure 1E), maximal OCR (Supplementary Figure 1F), ATP-linked OCR (Supplementary Figure 1G), SRC (Supplementary Figure 1H) and mitochondrial OCR (Figure 3D) indicating the requirement of autophagy for optimal induction of OXPHOS upon TLR-stimulation of pDC. Notably, 3-MA significantly reduced both IFNα secretion (Figure 3E) as well as expression of CD80 and PD-L1 upon pRNA-stimulation of pDCs (Figure 3F). Since, TLR7/8 stimulated pDC activation was prevented by pharmacological attenuation of OXPHOS, glutaminolysis and autophagy, we next asked whether the observed reduction was due to effect on cell viability. Analysis of cell viability revealed that BPTES, DON, 3-MA, ROT and AA did not affect viability of pDC alone or in combination with pRNA (Supplementary Figures 7, 8). Together, these data show that TLR7/8-stimulated pDC activation requires autophagy-supplemented glutaminolysis to fuel OXPHOS.

### TLR7/8 stimulated alterations in mitochondrial dynamics triggers glycolysis which is required for CD1c^+^ mDC activation

**Figure 4:**
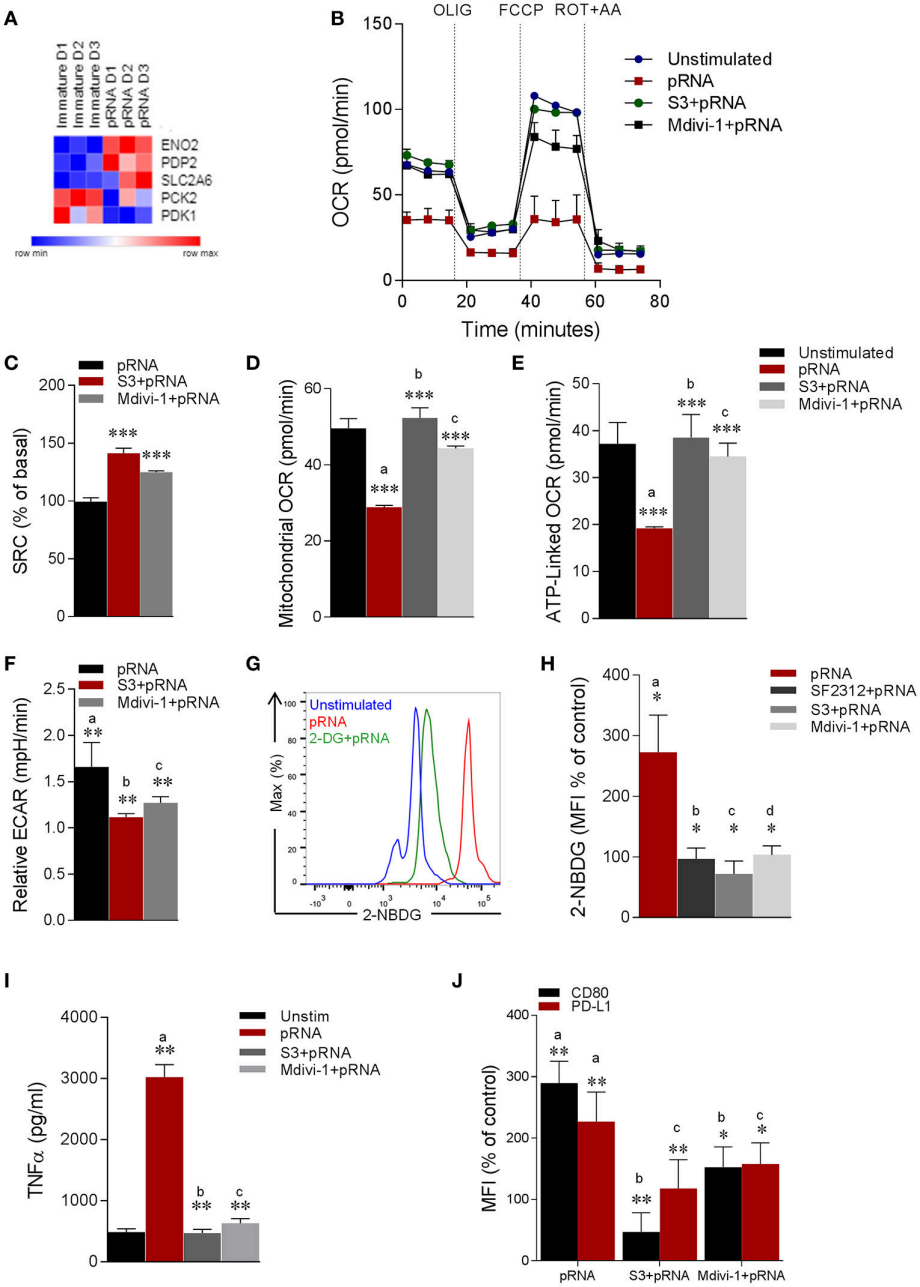
pRNA-stimulation alters mitochondrial morphology in CD1c^+^ mDC to induce glycolysis. **(A)** Heatmap showing expression of significantly changed genes which regulate glycolysis in CD1c^+^ mDC upon pRNA-stimulation for 6 h. Red color indicates increased expression while blue color shows decreased expression. **(B)** Mitochondrial fitness test of CD1c^+^ mDC stimulated with pRNA for 6 h in the presence or absence of 5 μM S3 or 1 μM Mdivi-1. Data represents mean ± SEM of three independent experiments. **(C–F)** Data was collected within same experiments as **(B)**, but is shown separately for better understanding. Data represents mean ± SEM of three independent experiments. ^*^p < 0.05; ^**^p < 0.01; ^***^p < 0.001 (Student's *t*-test). **(G)** Flow cytometry histograms of 2-NBDG stained CD1c^+^ mDC cells. **(H)** Percentage mean fluorescence intensity of cells stained with 2-NBDG. Data represents mean ± SEM of four independent experiments ^*^p < 0.05 (Student's *t*-test). **(I)** TNF-α levels on protein level were measured in the supernatant of the stimulated CD1c^+^ mDC cells stimulated for 6 h in the presence or absence of 5 μM S3 or 1 μM Mdivi-1. Data represents mean ± SEM of three independent experiments. ^**^p < 0.01 (Student's *t*-test). **(J)** Percentage mean fluorescence intensity of maturation markers (CD80 and PD-L1) in CD1c^+^ mDC cells stimulated for 6 h in the presence or absence of 5 μM S3 or 1 μM Mdivi-1. Data represents mean ± SEM of three independent experiments. ^*^p < 0.05; ^**^p < 0.01 (Student's *t*-test).

Our data show that TLR7/8-stimulation reduces expression of OXPHOS related genes and mitochondrial content in CD1c^+^ mDCs, which is associated with metabolic changes with a shift toward glycolysis (58) to compensate for the reduced activity of the respiratory chain to generate ATP (17). In this sense, we wondered whether mitochondrial alterations induced by TLR7/8-stimulation led to a metabolic shift in CD1c^+^ mDC. To this end, analysis of glycolysis related genes showed significant upregulation of *ENO2* (Figure 4A). *ENO2* encodes a dimeric enzyme, Enolase, which catalyzes the second last step in glycolysis i.e., interconverting 2-phosphoglycerate (2-PGA) and phosphoenolpyruvate (PEP) (59). Next, we monitored EFA in pRNA-stimulated CD1c^+^ mDC. We found that TLR7/8-stimulation significantly reduced OCR (Figure 4B; Supplementary Figure 2C). To test our hypothesis that mitochondrial fragmentation leads to induction of glycolysis in CD1c^+^ mDC upon TLR7/8-stimulation, we monitored EFA in the presence of S3 and Mdivi-1. Interestingly, S3 and Mdivi-1 significantly prevented the pRNA-induced decrease in OCR (Figure 4B; Supplementary Figures 2A–C), SRC (Figure 4C), mitochondrial OCR (Figure 4D) ATP-linked OCR (Figure 4E) and maximal OCR (Supplementary Figure 2D) in CD1c^+^ mDCs.

To investigate the induction of glycolysis, we monitored pRNA-induced ECAR in CD1c^+^ mDC. Importantly, pRNA stimulation significantly increased ECAR in CD1c^+^ mDC (Figure 4F). Of note, S3 and Mdivi-1 significantly reduced the pRNA-induced increase in ECAR (Figure 4F), indicating that indeed mitochondrial fragmentation induced by TLR7/8-stimulation leads to a shift toward glycolysis in CD1c^+^ mDC.

To further investigate the induction of glycolysis, we determined glucose uptake in CD1c^+^ mDCs upon TLR7/8-stimulation using 2-NBDG. Consistent with the increase in ECAR, pRNA-stimulation significantly increased the uptake of 2-NBDG in CD1c^+^ mDC, which could be prevented by glycolysis inhibitor, 2-DG (Figure 4G). Additionally, given the significant upregulation of *ENO2* upon pRNA-stimulation in CD1c^+^ mDC, we determined 2-NBDG uptake in the presence of a specific Enolase inhibitor, SF2312 (59). Consistently, the pRNA-induced 2-NBDG uptake in CD1c^+^ mDCs was significantly reduced in the presence of SF2312 (Figure 4H; Supplementary Figure 3). Similarly, S3 and Mdivi-1 treatment significantly reduced pRNA-induced 2-NBDG uptake (Figure 4H; Supplementary Figure 3A). Taken together, these data indicate that mitochondrial fragmentation induced by TLR7/8-stimulation leads to a shift toward glycolysis in CD1c^+^ mDC.

Next, we asked whether TLR7/8-stimulation induced alteration in mitochondrial dynamics are required for CD1c^+^ mDC activation. Importantly, pRNA stimulation significantly increased TNFα production, which was attenuated by S3 and Mdivi-1 (Figure 4I). Similarly, pRNA stimulation significantly upregulated maturation markers i.e., CD80 and PD-L1 on CD1c^+^ mDC, which were significantly inhibited by S3 and Mdivi-1 (Figure 4J). By comparison, we observed no effect of S3 and Mdivi-1 on pRNA-stimulated IFNα (Supplementary Figure 3E) and CD80 and PD-L1 in pDC (Supplementary Figure 3D). Collectively, these data indicate that TLR7/8-induced mitochondrial fragmentation is required for induction of glycolysis and immune response of CD1c^+^ mDC.

### TLR7/8-stimulation triggers BNIP3-dependent mitophagy in CD1c^+^ mDC

**Figure 5:**
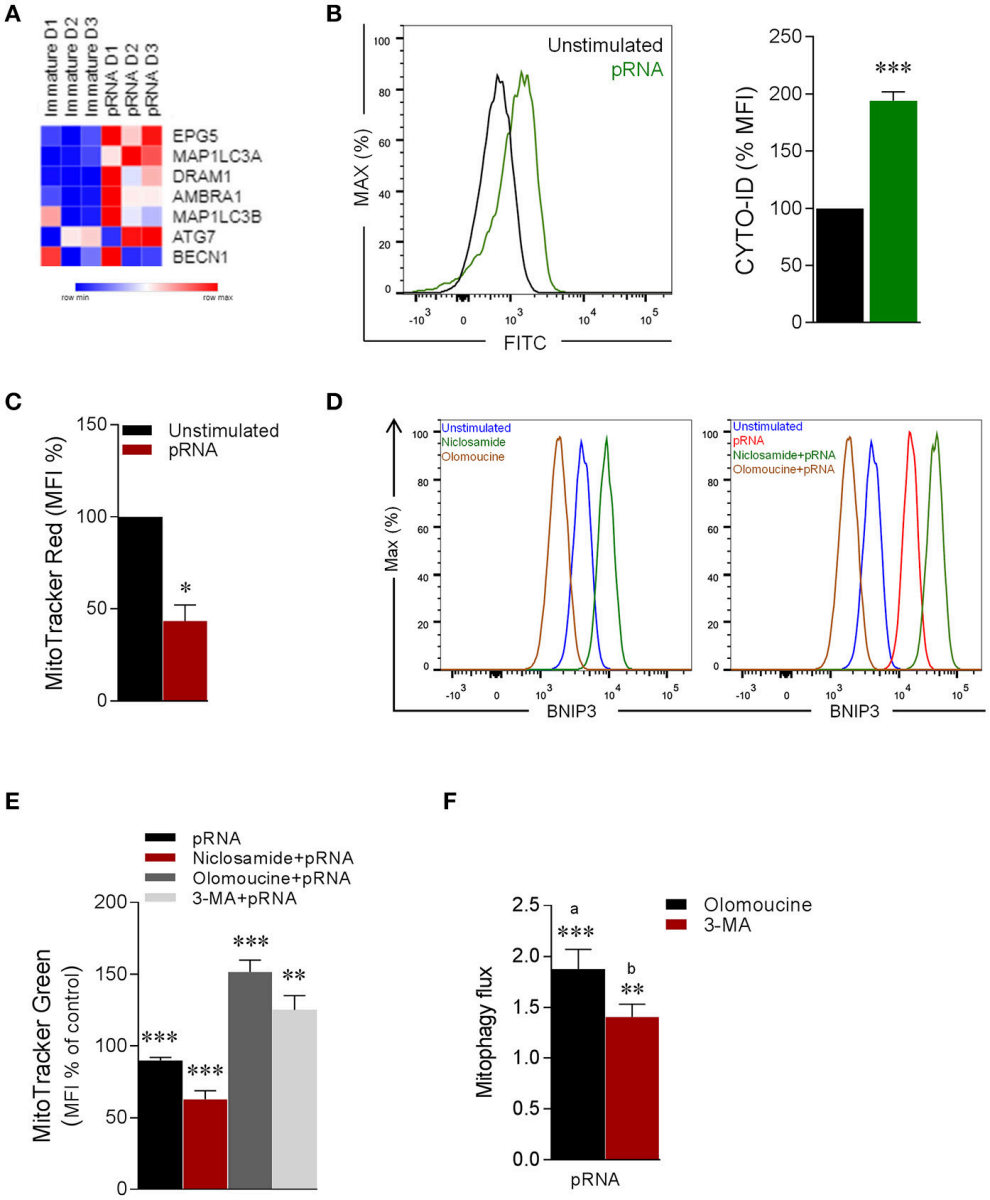
pRNA-stimulation triggers BNIP3-dependent mitophagy in CD1c^+^ mDC. **(A)** Heatmap showing expression of significantly changed genes which regulate autophagy in CD1c^+^ mDC upon pRNA-stimulation for 6 h. Red color indicates increased expression while blue color shows decreased expression. **(B)** Fluorescence intensity of autophagosomal marker CYTO-ID in pDC stimulated with pRNA for 6 h ^***^p < 0.001 (Student's *t*-test). **(C)** Percentage mean flouresence intensity of CD1c^+^ mDC cells stained with MitoTracker Red stimulated with pRNA for 6 h. Data represents mean ± SEM of three independent experiments. ^*^p < 0.05 (Student's *t*-test). **(D)** Flow cytometry histograms of BNIP3 in CD1c^+^ mDC cells in the presence or absence of 2 μM niclosamide or 10 μM olomoucine for 6 h. **(E)** Percentage mean fluorescence intensity of CD1c^+^ mDC cells stained with MitoTracker Green stimulated with pRNA for 6 h in the presence or absence of 2 μM niclosamide or 10 μM olomoucine or 25 μM 3-MA. Data represents mean ± SEM of three independent experiments ^**^p < 0.01; ^***^p < 0.001 (Student's *t*-test). **(F)** Mitophagy flux in CD1c^+^ mDC stimulated with pRNA for 6 h. Data represents mean ± SEM of three independent experiments ^**^p < 0.01; ^***^p < 0.001 (Student's *t*-test).

Mitophagy is a highly regulated autophagy process during which damaged mitochondria are degraded and removed from the cell (23, 60–62). Given the alteration in mitochondrial dynamics in CD1c^+^ mDC upon TLR7/8-stimulation, we hypothesize that mitophagy is induced in CD1c^+^ mDC. To this end, analysis of autophagy-related genes revealed that pRNA-stimulation significantly increased expression of *EPG5, MAP1LC3A, DRAM1* & *AMBRA1* (Figure 5A), indicating involvement of autophagy. Consistent with increased expression of autophagy-related genes, pRNA significantly increased autophagosomes in CD1c^+^ mDC (Figure 5B). Damaged mitochondria exhibit dissipated membrane potential, which is the initial trigger for mitophagy (22, 63). To test whether pRNA-stimulation affects mitochondrial membrane potential (Δψ) in CD1c^+^ mDC, we measured Δψ using MitoTracker Red CMXRos, a red-fluorescent dye which stains mitochondria in a membrane potential dependent manner (64). Importantly, pRNA-stimulation significantly induced Δψ depolarization in CD1c^+^ mDC (Figure 5C). Two distinct mitophagy pathways have been described. One engages ubiquitination of OMM proteins *via* the PINK1/Parkin-mediated pathway. Consequently, ubiquitinated proteins recruit autophagosomal membrane via specific receptors, which can recognize ubiquitin chains on mitochondrial proteins and LC3 at autophagosomal membrane (65). The other mitophagy pathway involves BNIP3, a Bcl-2 family member that regulates mitophagy by associating itself on the outer mitochondrial membrane (OMM) through C-terminal transmembrane domain and interacts with LC3 through its LC3-interacting region (LIR) domain located at N-terminal part (66–68). To determine which mitophagy pathway is involved upon TLR7/8-stimulation of CD1c^+^ mDC, the gene expression data were examined. Interestingly, PINK1 did not significantly change upon pRNA stimulation, whereas BNIP3 was significantly increased in CD1c^+^ mDC upon pRNA-stimulation (Supplementary Figure 2E).

To specify the involvement of BNIP3, the effect of olomoucine, a transcriptional inhibitor of BNIP3 (69) on TLR7/8-induced mitophagy in CD1c^+^ mDC was examined. Olomoucine significantly reduced steady state BNIP3 (Figure 5D) and the pRNA-induced increase of BNIP3 in CD1c^+^ mDC (Figure 5D). Niclosamide is a transcriptional inhibitor of S100A4 (70), which is transcriptional repressor of of BNIP3 (71). Niclosamide increased BNIP3 expression in CD1c^+^ mDC (Figure 5D). To quantitatively asses mitophagy in CD1c^+^ mDC cells, we employed flow cytometry based method (72). This approach is suitable to robustly assess mitophagy without need to perform traditional fluorescence microscopy of mitochondrial-autophagosome colocalization in BNIP3 transfected cells, in order to avoid transfection and prolonged culture-induced cell death in rare human CD1c^+^ mDC cells. The reversal in alteration in MitoTracker upon mitophagy inhibitors (i.e., olomoucine and 3-MA) indicates induction of mitophagy and can be used to calculate mitophagic flux (72). Of note, loss of pRNA-induced mitochondrial content in CD1c^+^ mDC cells was significantly potentiated by niclosamide, which augments BNIP3 expression (Figure 5E). On other hand, loss of pRNA-induced mitochondrial content in CD1c^+^ mDC cells was significantly reversed by olomoucine and 3-MA (Figure 5E) indiacating induction of mitophagy. Furthermore, analysis of mitophagic flux, revealed that pRNA stimulation significantly increased mitophagic flux in CD1c^+^ mDC (Figure 5F). This data indicates that TLR7/8-stimulation triggers BNIP3-dependent mitophagy in CD1c^+^ mDC cells.

### TLR7/8-stimulated BNIP3-dependent mitophagy is indispensable for induction of glycolysis and activation of CD1c^+^ mDC

**Figure 6:**
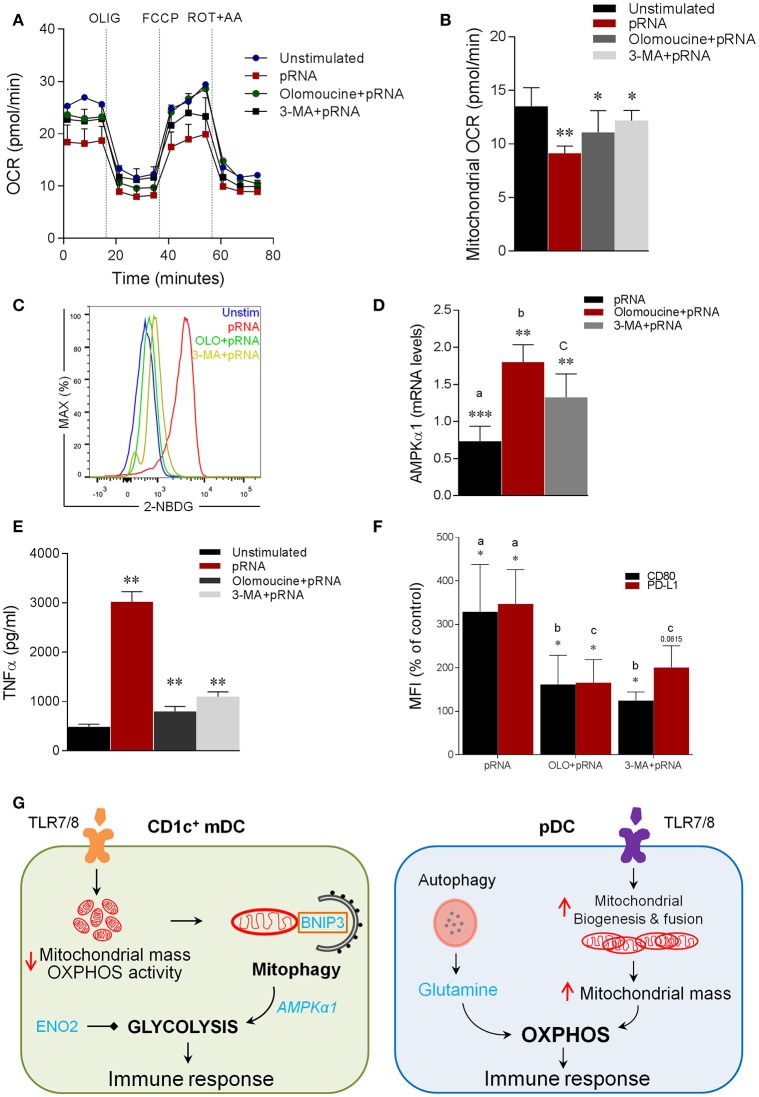
Mitophagy is indispensable for induction of glycolysis and activation of CD1c^+^ mDC **(A)** Mitochondrial fitness test of CD1c^+^ mDC stimulated with pRNA for 6 h in the presence or absence of 10 μM olomoucine or 25 μM 3-MA. Data represents mean ± SEM of three independent experiments. **(B)** Data was collected within same experiments as **(A)** but is shown separately for better understanding. Data represents mean ± SEM of three independent experiments. ^*^p < 0.05; ^**^p < 0.01 (Student's *t*-test). **(C)** Flow cytometry histograms of 2-NBDG stained CD1c^+^ mDCs stimulated with pRNA pDC for 6 h. **(D)** *AMPK*α*1* mRNA levels were analyzed after 6 h of pRNA stimulation by (qPCR) and normalized to β-actin expression by using the 2^ΔΔ*CT*^ method. Data represents Mean±SEM of three independent experiments ^**^p < 0.01; ^***^p < 0.001 (Student's *t*-test). **(E)** TNF-α levels on protein level were measured in the supernatant of the CD1c^+^ mDC stimulated for 6 h. Data represents mean ± SEM of three independent experiments ^**^p < 0.01 (Student's *t*-test). **(F)** Percentage mean flouresence intensity of maturation markers (CD80 and PD-L1) in CD1c^+^ mDC cells stimulated for 6 h in the presence or absence of 10 μM olomoucine or 25 μM 3-MA. Data represents mean ± SEM of three independent experiments ^*^p < 0.05 (Student's *t*-test). **(G)** Proposed model of human DC-subsets activation via TLR7/8 agonist (CD1c^+^ mDC) TLR-stimulation reduces mitochondrial content, OXPHOS activity and induces glycolysis in CD1c^+^ mDC. TLR-stimulation in CD1c^+^ mDCs results in depolarized mitochondrial membrane potential (Δψ) and triggers BNIP3-dependent mitophagy which is required for induction of glycolysis and activation of CD1c^+^ mDC (pDC) TLR-stimulation increases OXPHOS and mitochondrial content as result of increased protein levels of Mfn2 and PGC1α in pDC. Moreover, TLR-stimulation in pDC increases intracellular glutamine in an autophagy-dependent manner. TLR-induced glutaminolysis fuels increases OXPHOS in pDCs which are indispensable for pDC activation.

Notably, mitophagy has been reported to be required for glycolytic switch in tumor cells (73). Given, the metabolic reprogramming toward glycolysis in CD1c^+^ mDC upon TLR7/8 stimulation, we next asked whether BNIP3-dependnet mitophagy is required for induction of glycolysis in CD1c^+^ mDC. To investigate this, we monitored EFA in the presence or absence of olomoucine and 3-MA in CD1c^+^ mDC. Intriguingly, olomoucine and 3-MA significantly prevented the pRNA-induced decrease in OCR (Figure 6A; Supplementary Figure 4A), mitochondrial OCR (Figure 6B), ATP-linked OCR (Supplementary Figure 4B), maximal OCR (Supplementary Figure 4C) and SRC (Supplementary Figure 4D) in CD1c^+^ mDCs. Moreover, olomoucine and 3-MA prevented pRNA-stimulated uptake of 2-NBDG (Figure 6C). These experiments indicate that BNIP3-dependent mitophagy is indispensible for induction of glycolysis in CD1c+ mDC upon TLR7/8 stimulation. To elucidate the mechanism underlying BNIP3 regulation of glycolysis, we examined the involvement of AMPK, which is key regulator of metabolic homeostasis (74). pRNA stimulation significantly reduced *AMPK1*α mRNA levels in CD1c^+^ mDC, which were significantly rescued by olomoucine and 3-MA (Figure 6D). Interestingly, mitophagy inhibition attenuated TLR7/8-stimulated immune response in CD1c^+^ mDC, as olomoucine and 3-MA significantly reduced pRNA-stimulated TNFα levels (Figure 6E). Moreover, the pRNA-induced increase in maturation markers CD80 and PD-L1 was significantly decreased in the presence of olomoucine and 3-MA (Figure 6F). By comparison, olomoucine had no effect on pRNA stimulated IFNα (Supplementary Figure 3E) and CD80 and PD-L1 in pDC (Supplementary Figure 3D). Of note, 2-DG, SF2313, Mdivi-1, S3, 3-MA, olomoucine and niclosamide did not affect viability of CD1c^+^ mDC alone or in combination with pRNA (Supplementary Figures 5, 6). Collectively, these data suggest that TLR7/8-stimulated BNIP3-dependent mitophagy is crucial for induction of glycolysis, which contributes to CD1c^+^ mDC activation.

## Discussion

Changes in metabolism following TLR stimulation are indispensable for DC activation. However, the metabolic signature generated in naturally occurring human DCs in response to TLR-stimulation is not known in detail. Herein, we investigated TLR-induced metabolic changes in two human blood DC-subsets, CD1c^+^ mDC and pDC. Our data show that TLR stimulation results in a differential mitochondrial rewiring in pDC and CD1c^+^ mDC. We have focused on mitochondria as metabolic hubs critical for signals downstream of innate receptors in myeloid cells (75). Promotion of mitochondrial fusion results in increased OXPHOS activity *via* formation of supercomplexes (76). Supercomplex reorganization in macrophages is also driven by innate sensing of microbes, regulating macrophage cytokine production (77). Conversely, mitochondrial fission results in decreased OXPHOS activity and induction of glycolysis (21). Interestingly, mitochondrial dynamics play an important role in differentiation and migration of immature DC (78). Mitochondrial fusion proteins are upregulated during differentiation of bone marrow progenitors to immature DC. Mitochondrial fusion-related proteins i.e., Mfn2 and Opa1 have been shown to be required for migration of immature DC (78).

Here, we investigated the role of mitochondrial dynamics in regulating immune function of human DC subsets. We find that stimulation of pDCs with TLR7/8 agonist increases expression of PGC1α and Mfn2, which suggests increase in mitochondrial mass. Indeed, we observed that TLR7/8-stimulation resulted in increased mitochondrial mass in pDC, as demonstrated by MitoTracker Green and Porin levels. Moreover, PGC-1α positively regulates mitochondrial fusion by stimulating Mfn2 expression *via* targeting the Mfn2 promoter in an ERRα-binding element-dependent manner (79). Importantly, increased Mfn2 expression results in increased glucose oxidation and expression of OXPHOS complex I, IV and V (80). Consistently, we observed increased expression of OXPHOS related genes and protein levels of NDUFA10 upon TLR7/8-stimulation in pDC, indicating upregulation of OXPHOS. Taken together, these data indicate that TLR7/8 stimulation increases mitochondrial fusion, mass and increased OXPHOS activity in pDC. Conversely, pRNA stimulation of CD1c^+^ mDCs results in increased expression of Drp1, which contributes to mitochondrial fission (81, 82), which lead to decrease in mitochondrial mass as shown by decreased levels of MitoTracker Green and Porin. Mitochondrial fission promotes a shift to aerobic glycolysis (58, 83, 84). Our data shows that TLR-stimulation leads to increased glycolysis in CD1c^+^ mDC. Increased expression of Drp1 together with decreased expression of NDUFA10 and mitochondrial mass, in CD1c^+^ mDC indicates induction of mitochondrial fission, which is linked to glycolysis (21, 85, 86). Intriguingly, Drp1 has been demonstrated to be required for the activation of bone marrow-derived DCs upon LPS-stimulation (87). It has been reported that TLR-stimulated metabolic reprogramming is required to meet the energy demand for the activation process in DC (14, 16, 88). Of note, our data show that mitochondrial dynamics modulate expression of inflammatory mediators (i.e., TNFα, CD80, and PD-L1) in human DC-subsets. Our data highlights the importance of mitochondrial remodeling in innate sensing.

Both fission and fusion proteins also play a key role in mitophagy regulation. Upon stress, Drp1 specifically splits a mitochondrion into a healthy fraction and a damaged fraction, to promote degradation of damaged fraction *via* mitophagy (23). To this end, our data show that TLR-stimulation induces BNIP3-dependent mitophagy in CD1c^+^ mDC. Additionally, we demonstrate that TLR-stimulated mitophagy and glycolysis are essential for CD1c^+^ mDC activation. We further demonstrate induction of *Enolase*-dependent glycolysis in CD1c^+^ mDC upon TLR-stimulation. Consistently, ENO2 inhibition impairs CD1c^+^ mDC maturation and activation. These results implicate increased glycolysis for proficient antigen processing and presentation by CD1c^+^ mDC to induce a robust immune response. Previously, *Chlamydia* infection was shown to increase mitochondrial permeability in parallel with mitochondrial remodeling in Enolase1 (ENO1)-dependent manner in mouse bone marrow-derived DCs (89). Intriguingly, BNIP3-dependent mitophagy contributes to mitochondrial elimination during polarization toward pro-inflammatory and glycolytic macrophages (90).

Of note, metabolic reprogramming toward glycolysis is regulated by mitophagy, as mitophagy inhibition reduced expression of glycolysis regulators e.g., *PFKFB3, HK2, GAPDH*, and *PKM2* (90). Therefore, it is conceivable that BNIP3-dependent mitophagy similarly controls glycolysis regulators in CD1c^+^ mDC. We found that BNIP3 regulates transcriptional activity of *AMPK*α*1*. AMPK is a negative regulator of aerobic glycolysis (91). Intriguingly, AMPK activation has been reported to antagonize glycolytic switch in DCs (14). Our data shows that TLR7/8-stimulation decreases *AMPK*α*1* which can be restored upon BNIP3 inhibition. In contrast, loss of BNIP3 has been reported to reduce AMPK activity in liver (92). However, recent studies have demonstrated that AMPK activation can also be regulated *via* reactive oxygen species (ROS) (93). Of note, mitophagy regulates ROS (19), which in turn can act as transcription factor to control gene expression (94). Therefore, it is possible that BNIP3 inhibition reduces mitophagy, which in turn suppresses ROS levels to modulate *AMPK*α*1* in CD1c^+^ mDC. Glycolysis is also required for canonical activation of the inflammasome in macrophages (95, 96). Interestingly, TLR-stimulation has been shown to induce inflammasome activation in CD1c^+^ mDC (97). Intriguingly, autophagy negatively regulates NLRP3 inflammasome activation in macrophages and bone marrow derived DC (98, 99). Moreover, mitophagy prevents hyper-inflammation triggered by NLRP3 inflammasome activation in macrophages (100). Our data show that mitophagy is indispensable for CD1c^+^ mDC activation. Collectively, our data suggest a scenario in which TLR-stimulation results in mitochondrial fission leading to induction of mitophagy, which in turn regulates glycolysis via *AMPK*α*1* to activate CD1c^+^ mDC.

It has been demonstrated that autophagy is required for production of type I IFNs in pDC following TLR7 signaling *in vitro* and *in vivo* (48–52). To this end, TLR7-stimulated autophagy deficient pDCs are unable to produce IFNα, in comparison to their autophagy proficient counterparts (49, 48). We here demonstrate that autophagy serves to provide glutamine to fuel OXPHOS in pDC upon TLR-stimulation, similar to mechanisms previously shown in tumor cells (54–56). Our data show that TLR-stimulation in pDCs increases cellular glutamine levels in an autophagy dependent-manner. Additionally, autophagy inhibition abrogates glutamine fueled OXPHOS in pDCs upon TLR stimulation. Autophagy is involved in regulating several DC functions e.g., DC maturation, antigen presentation, cytokine production, DC migration and T-cell activation (101). Herein, we provide novel insight into pDC innate sensing mechanism by providing link between autophagy and type I IFN production by demonstrating that autophagy serves to provide glutamine, which is required for IFNα production. Conversely, selective autophagy i.e., mitophagy is required for induction of glycolysis *via* AMPKα1 regulation. Thus, our data provides novel mechanistic insight in differential role of autophagy in human DC subsets that can lead to immunostimulatory phenotype.

TLR stimulation triggers a shift in metabolism toward aerobic glycolysis, in human mDCs and mouse bone-marrow derived DCs (BMDCs), which is indispensable for the immune effector function and survival of DCs (14, 15, 102, 103). This shift toward glycolysis is required to support the metabolic requirements coupled with increased protein synthesis, which contributes to DC immunogenicity. This TLR-induced surge in glycolysis initiates *de novo* fatty acid synthesis through glucose-dependent citrate metabolism, which sustains the synthesis and secretion of inflammatory cytokines (103, 104). Furthermore, disrupting the glucose-to-citrate pathway reduces DC maturation, cytokine secretion and in turn T cell stimulatory capacity. Influenza virus (flu), Rhinovirus (RV) and a TLR7 agonist induce early glycolysis in human pDC, which is required for type I IFN production and upregulation of HLA-DR, CD80, CD86 (105). However, the generated type I IFN can in turn signal through IFNAR in a paracrine way to trigger FAO and OXPHOS in pDC (16). We find increased glutamine levels after TLR-stimulation in pDC. Of note, glutaminase inhibition in pDCs attenuated OXPHOS, suggesting that glutaminolysis drives OXPHOS induction in response to TLR stimulation in pDC. The requirement of glutamine for various immune effector functions has been demonstrated, e.g., LPS-driven inflammatory response in succinate-dependent anaplerosis (106, 107). However, these reports show that activity of glutamine depends on glycolysis. In contrast, it has also been reported that glutamine drives glucose-independent TCA cycle (108). Additionally, glutamine has been demonstrated to be required for trained immunity in monocytes (109), for activated T cells to fuel metabolism (110) and cytokine production by lymphocytes and macrophages (111). Tumor associated M2-like macrophages utilize glutamine for TCA cycle activity, which is required for M2 polarization (112). Moreover, tumor associated macrophages in glioblastoma show increased glutamate transport and metabolism (113). Intriguingly, glutaminolysis has been reported to be dispensable for mouse bone marrow-derived DCs cultured in the presence of GM-CSF for activation upon TLR-stimulation (114). Moreover, it is possible that type I IFN paracrine signaling in TLR-stimulated pDC contributes to the induction of fatty acid oxidation, as shown for CpG stimulated murine pDC (16).

Our study provides several novel insights into TLR-stimulated metabolic adaptations in human DC subsets. Our data demonstrate that different DC-subsets engage distinct metabolic adaptations in a mitochondrial dynamics-dependent manner following TLR stimulation. Furthermore, our study provides novel mechanistic insights in human DC-subset metabolism by demonstrating the involvement of mitophagy dependent-glycolysis in CD1c^+^ mDC and autophagy supplemented glutaminolysis for OXPHOS in pDC (Figure 6G). As metabolic manipulation results in modulation of DC activation, our results may have important implications in development of DC-based therapies.

## Author contributions

FB and TM performed the experiments. FB analyzed the data. FB, DS, and IdV wrote the manuscript. IdV supervised the research.

### Conflict of interest statement

The authors declare that the research was conducted in the absence of any commercial or financial relationships that could be construed as a potential conflict of interest. The handling Editor declared a past co-authorship with one of the authors DS.

## Supplementary material

Supplementary Figure 1 **(A–D)** Data were collected within the same experiments as Figure 2C but are shown separately for clarity. Data represents mean ± SEM of three independent experiments. ^***^*p* < 0.001 (Student's *t*-test). **(E–H)** Data were collected within the same experiments as Figure 3C but are shown separately for clarity. Data represents mean ± SEM of three independent experiments. ^**^*p* < 0.01; ^***^*p* < 0.001 (Student's *t*-test).

Supplementary Figure 2 **(A–D)** Data were collected within the same experiments as Figure 4B but are shown separately for clarity. Data represent mean ± SEM of three independent experiments. ^***^*p* < 0.001 (Student's *t*-test). **(E)** Relative gene expression of mitophagy related genes in CD1c^+^ mDC. Data represents mean ± SEM of three independent experiments. ^*^*p* < 0.05; ^**^*p* < 0.01 (Student's *t*-test).

Supplementary Figure 3 **(A)** Depicted is the mean fluorescence intensity of cells stained with 2-NBDG as percentage of the mean fluorescence intensity of control cells ± SEM of four independent experiments ^*^*p* < 0.05 (Student's *t*-test). **(B)** TNF-α levels on protein level were measured in the supernatant of the CD1c^+^ mDC stimulated for 6 h in the presence or absence of 10 nM rotenone or 10 nM antimycin A. Data represents mean ± SEM of three independent experiments ^**^*p* < 0.01 (Student's *t*-test). **(C)** Percentage mean fluorescence intensity of maturation markers (CD80 and PD-L1) in CD1c^+^ mDCs stimulated for 6 h in the presence or absence of 10 nM rotenone or 10 nM antimycin A. Data represents mean ± SEM of three independent experiments. ^**^*p* < 0.01 (Student's *t*-test). **(D)** IFN-α levels on protein level were measured in the supernatant of the pDC stimulated for 6 h in the presence or absence of either 5 μM S3 or 1 μM Mdivi-1 or 10 μM olomycine. Data represents mean ± SEM of three independent experiments ^***^*p* < 0.001 (Student's *t*-test). **(E)** Percentage mean fluorescence intensity of maturation markers (CD80 and PD-L1) in pDC stimulated for 6 h in the presence or absence of either 5 μM S3 or 1 μM Mdivi-1 or 10 μM olomycine. Data represents mean ± SEM of three independent experiments. ^**^*p* < 0.01; ^*^*p* < 0.05 (Student's *t*-test).

Supplementary Figure 4 **(A–D)** Data was collected within same experiments as 6A, but is shown separately for better understanding. Data represents mean ± SEM of three independent experiments. ^*^*p* < 0.05; ^**^*p* < 0.01; ^***^*p* < 0.001 (Student's *t*-test).

Supplementary Figure 5 CD1c^+^ mDC were stimulated with pRNA for 12 h in the presence or absence of 5 mM 2-DG or 500 nM SF2312 or 1 μM Mdivi-1. CD1c^+^ mDCs were stained with Fixable Viability Dye eFluor™ 780.

Supplementary Figure 6 CD1c^+^ mDC were stimulated with pRNA for 12 h in the presence or absence of 5 μM S3 or 25 μM 3-MA or 10 μM olomoucine or 2 μM niclosamide. CD1c^+^ mDCs were stained with Fixable Viability Dye eFluor™ 780.

Supplementary Figure 7 PDC were stimulated with pRNA for 12 h in the presence or absence of 5 μM BPTES or 10 μM DON or 25 μM 3-MA. pDCs were stained with Fixable Viability Dye eFluor™ 780.

Supplementary Figure 8 PDC were stimulated with pRNA for 12 h in the presence or absence of 10 nM rotenone or 10 nM antimycin A. pDCs were stained with Fixable Viability Dye eFluor™ 780.

## Abbreviations

PGC1α: peroxisome proliferator-activated receptor gamma coactivator 1-alpha
BNIP3: BCL2 interacting protein 3
Mfn1/2: mitofusin 1/2
Drp1: dynamin-related protein
ENO2: enolase
BPTES, bis-2-(5-phenylacetamido-1, 3: 4-thiadiazol-2-yl)ethyl sulfide
3-MA: 3-methyladenine
ROT: rotenone
AA: antimycin A
OXPHOS: oxidative phosphorylation
ETC: electron transport chain
pDC: plasmacytoid dendritic cell
CD1c^+^ mDC: CD1c^+^ myeloid dendritic cells
2-NBDG, 2-(N-(7-nitrobenz-2-oxa-1: 3-diazol-4-yl)amino)-2-deoxyglucose.
