# Supplementary figures and images for: Human Dendritic Cell Subsets Undergo Distinct Metabolic Reprogramming for Immune Response
Source: Front Immunol. 2018 Nov 1;9:2489. doi: 10.3389/fimmu.2018.02489 (PMC6230993; doi:10.3389/fimmu.2018.02489)

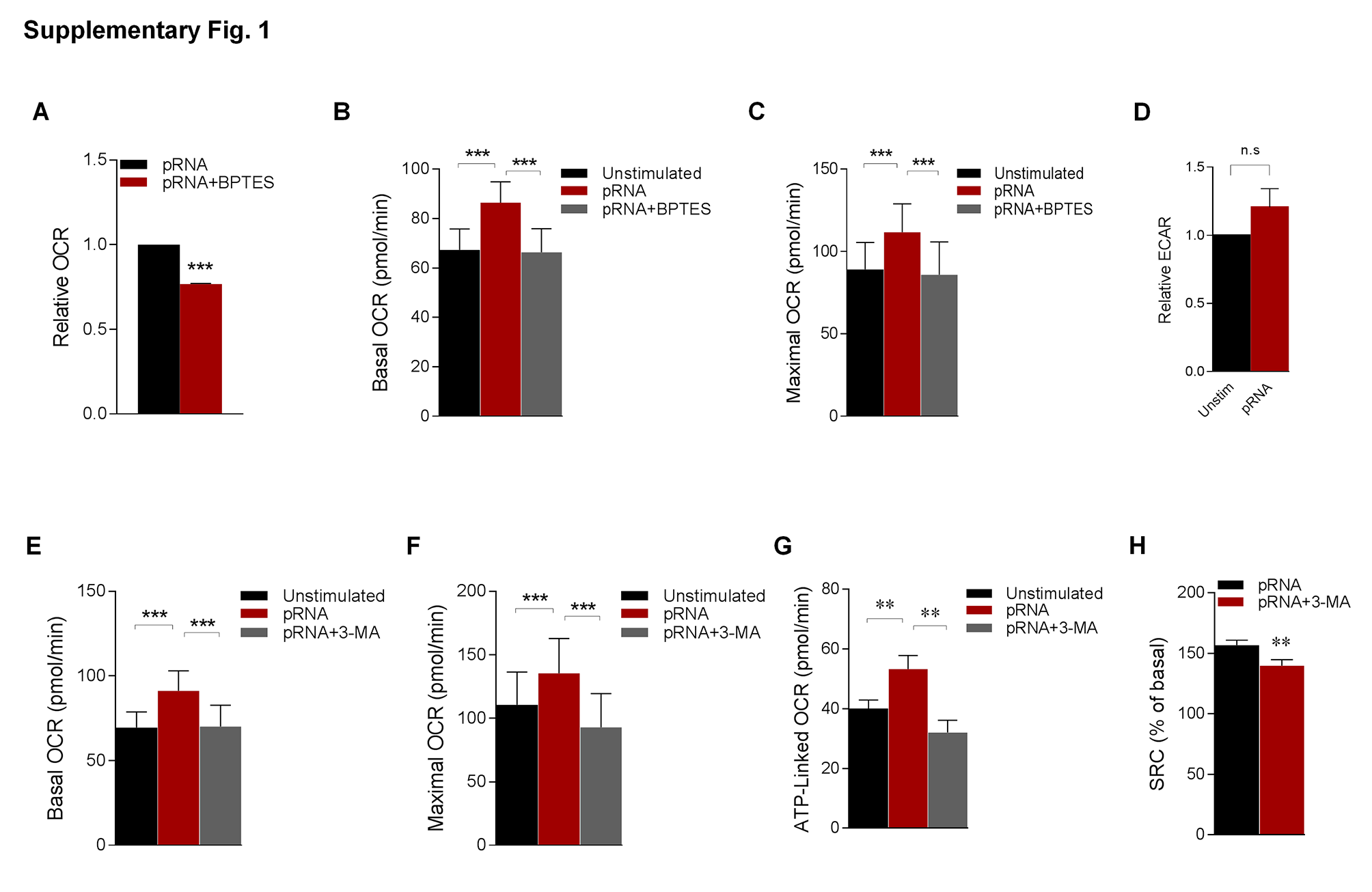

Supplement: Supplementary Figure 1 — (A–D) Data were collected within the same experiments as Figure 2C but are shown separately for clarity. Data represents mean ± SEM of three independent experiments. ***p < 0.001 (Student's t-test). (E–H) Data were collected within the same experiments as Figure 3C but are shown separately for clarity. Data represents mean ± SEM of three independent experiments. **p < 0.01; ***p < 0.001 (Student's t-test). [file Image_1.TIF]

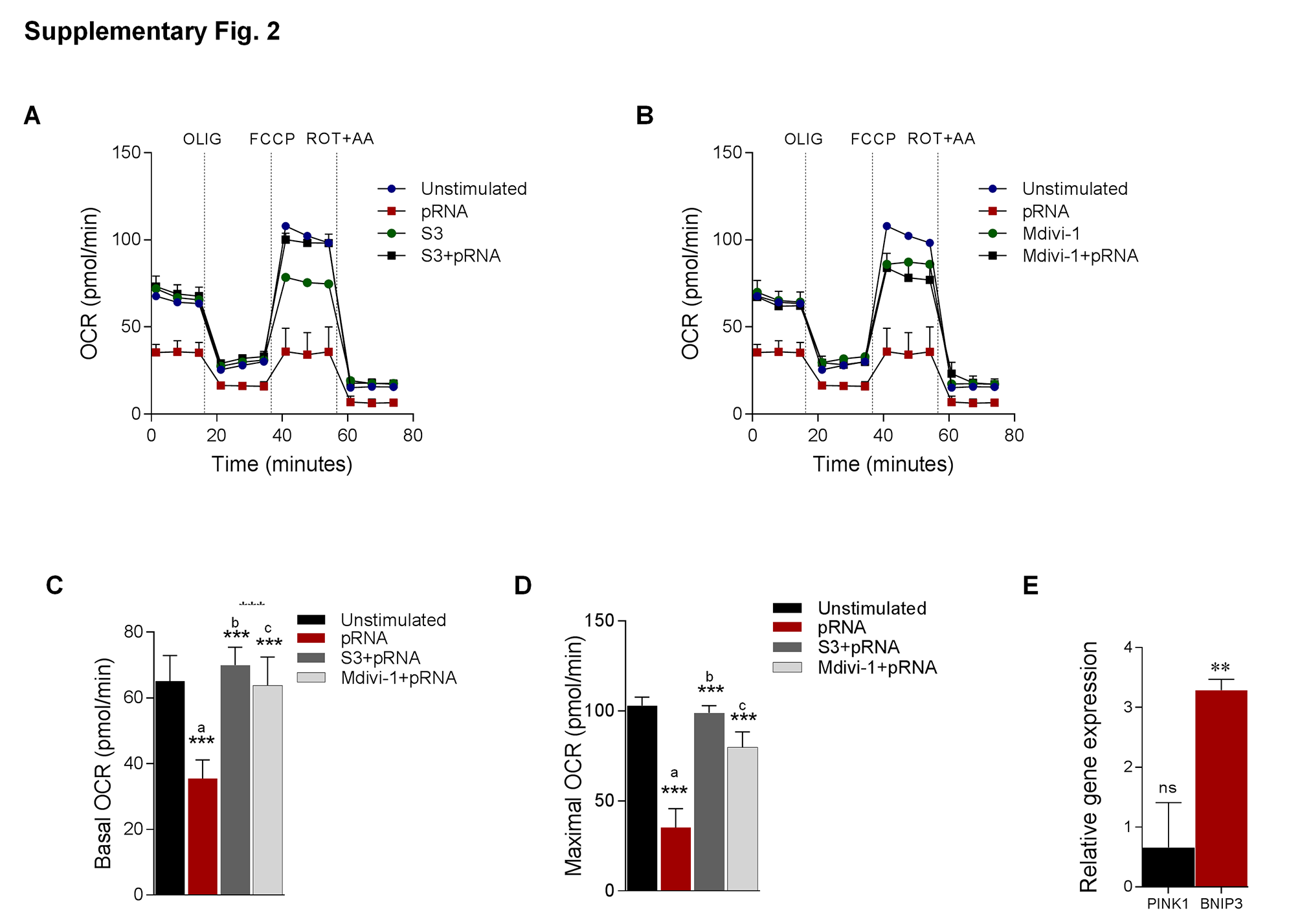

Supplement: Supplementary Figure 2 — (A–D) Data were collected within the same experiments as Figure 4B but are shown separately for clarity. Data represent mean ± SEM of three independent experiments. ***p < 0.001 (Student's t-test). (E) Relative gene expression of mitophagy related genes in CD1c+ mDC. Data represents mean ± SEM of three independent experiments. *p < 0.05; **p < 0.01 (Student's t-test). [file Image_2.TIF]

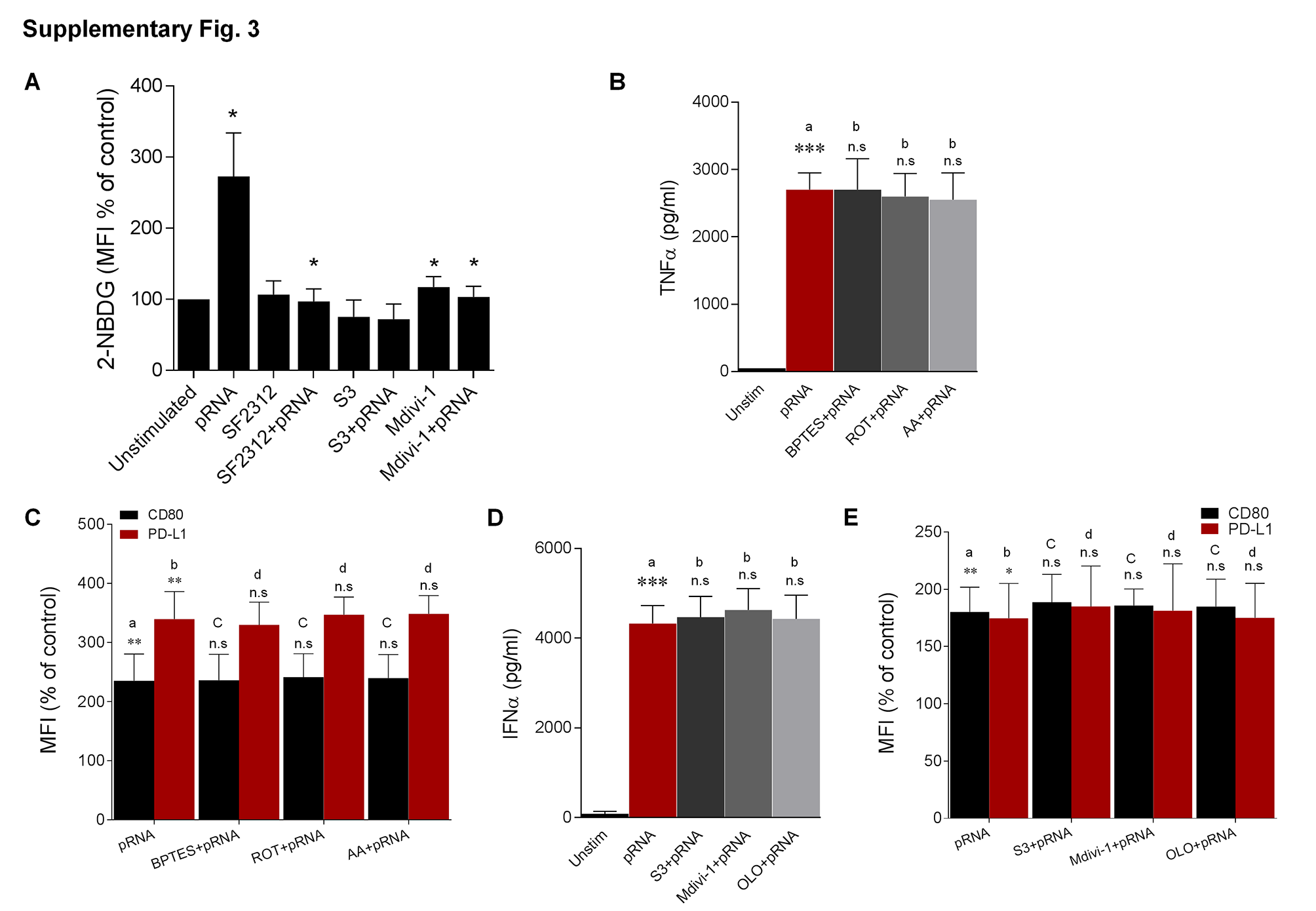

Supplement: Supplementary Figure 3 — (A) Depicted is the mean fluorescence intensity of cells stained with 2-NBDG as percentage of the mean fluorescence intensity of control cells ± SEM of four independent experiments *p < 0.05 (Student's t-test). (B) TNF-α levels on protein level were measured in the supernatant of the CD1c+ mDC stimulated for 6 h in the presence or absence of 10 nM rotenone or 10 nM antimycin A. Data represents mean ± SEM of three independent experiments **p < 0.01 (Student's t-test). (C) Percentage mean fluorescence intensity of maturation markers (CD80 and PD-L1) in CD1c+ mDCs stimulated for 6 h in the presence or absence of 10 nM rotenone or 10 nM antimycin A. Data represents mean ± SEM of three independent experiments. **p < 0.01 (Student's t-test). (D) IFN-α levels on protein level were measured in the supernatant of the pDC stimulated for 6 h in the presence or absence of either 5 μM S3 or 1 μM Mdivi-1 or 10 μM olomycine. Data represents mean ± SEM of three independent experiments ***p < 0.001 (Student's t-test). (E) Percentage mean fluorescence intensity of maturation markers (CD80 and PD-L1) in pDC stimulated for 6 h in the presence or absence of either 5 μM S3 or 1 μM Mdivi-1 or 10 μM olomycine. Data represents mean ± SEM of three independent experiments. **p < 0.01; *p < 0.05 (Student's t-test). [file Image_3.TIF]

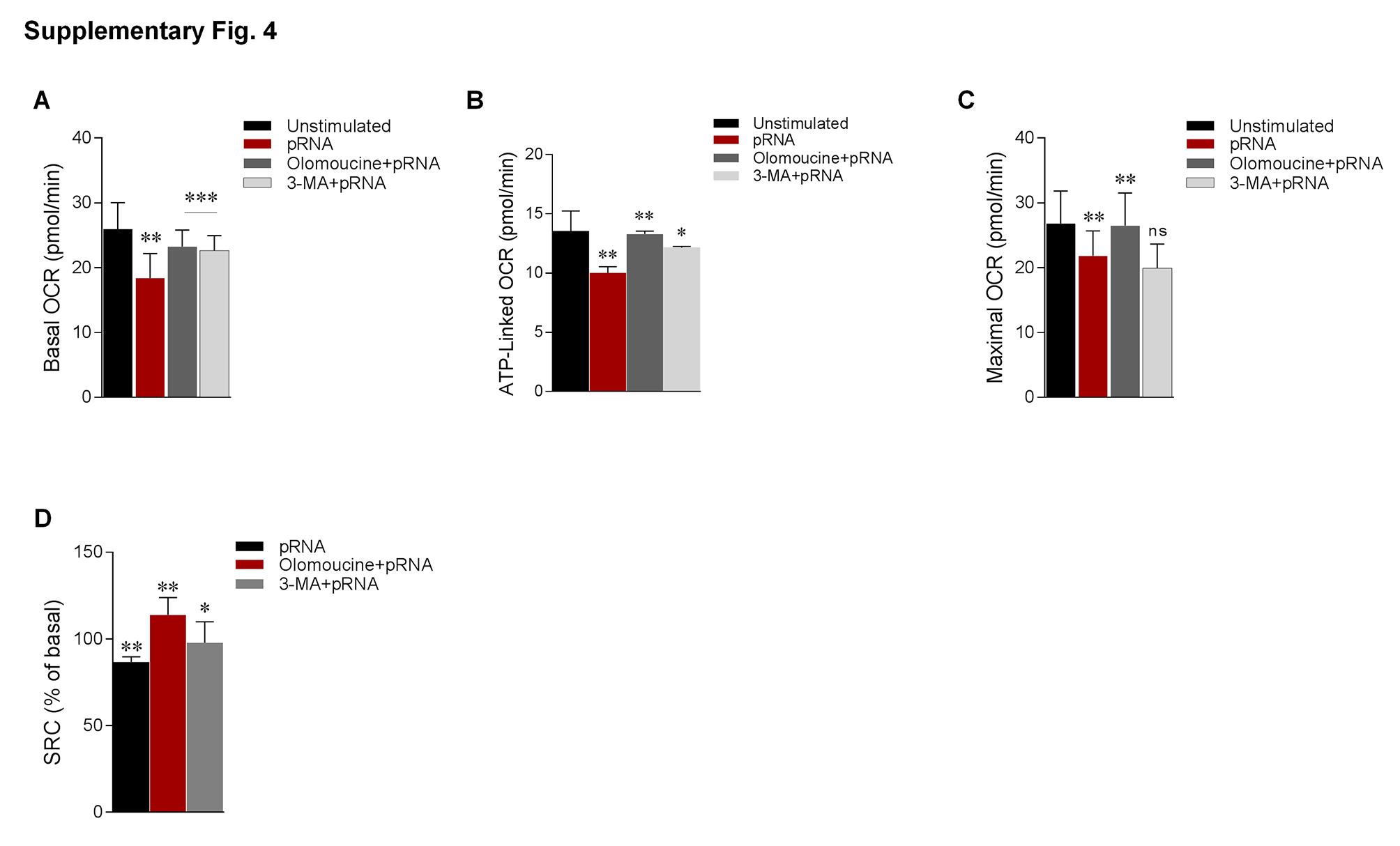

Supplement: Supplementary Figure 4 — (A–D) Data was collected within same experiments as 6A, but is shown separately for better understanding. Data represents mean ± SEM of three independent experiments. *p < 0.05; **p < 0.01; ***p < 0.001 (Student's t-test). [file Image_4.TIF]

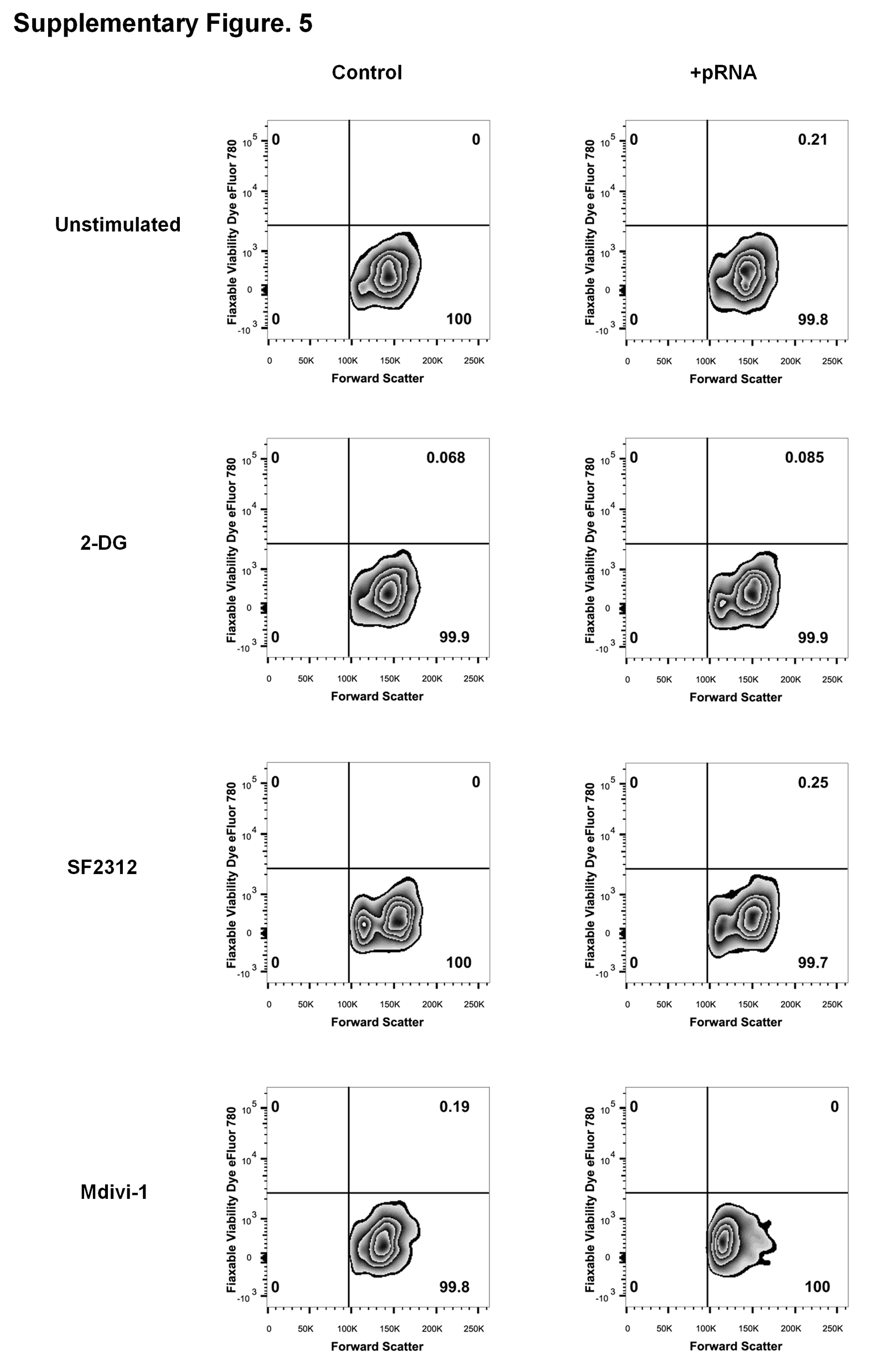

Supplement: Supplementary Figure 5 — CD1c+ mDC were stimulated with pRNA for 12 h in the presence or absence of 5 mM 2-DG or 500 nM SF2312 or 1 μM Mdivi-1. CD1c+ mDCs were stained with Fixable Viability Dye eFluor™ 780. [file Image_5.TIF]

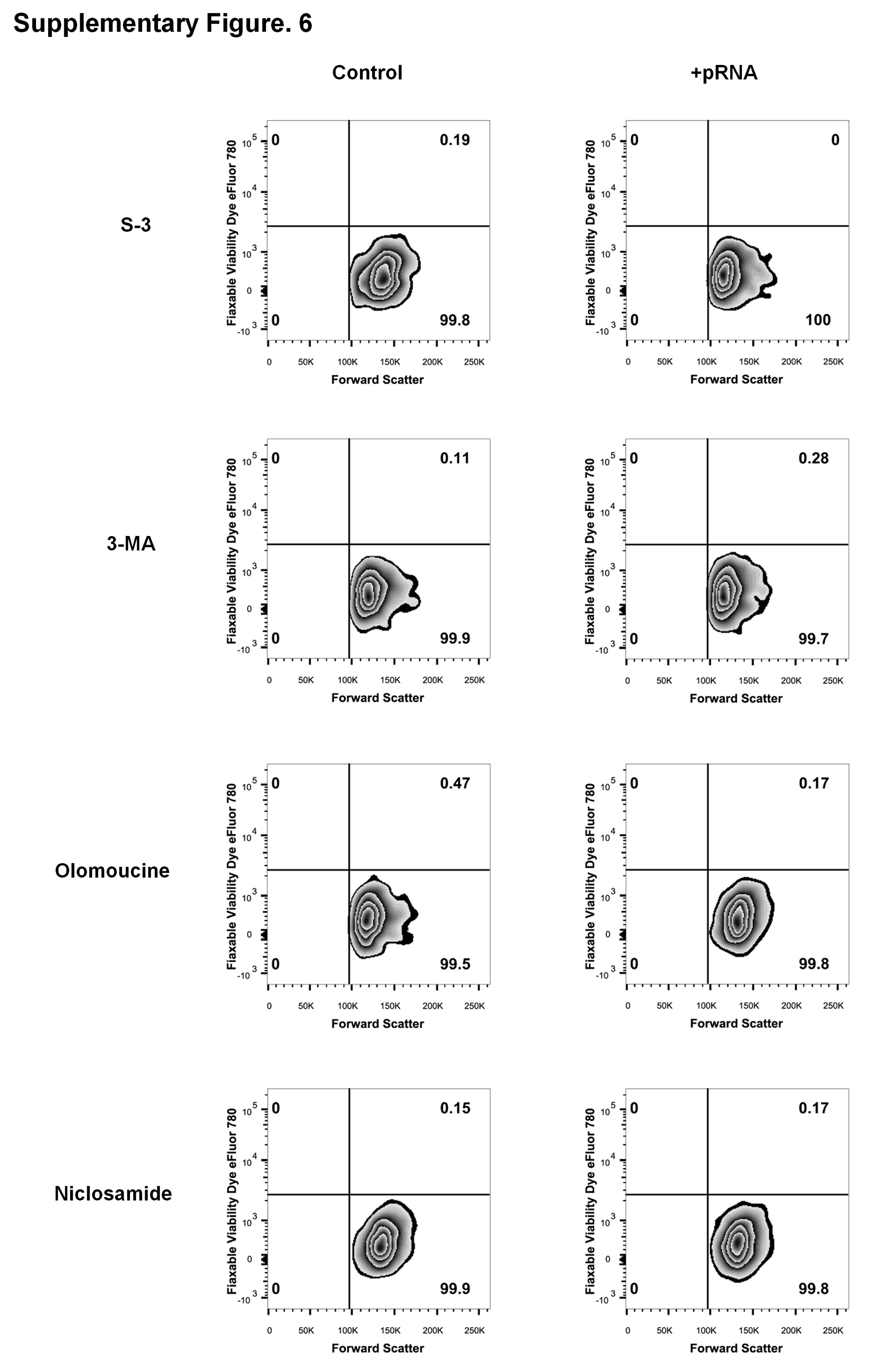

Supplement: Supplementary Figure 6 — CD1c+ mDC were stimulated with pRNA for 12 h in the presence or absence of 5 μM S3 or 25 μM 3-MA or 10 μM olomoucine or 2 μM niclosamide. CD1c+ mDCs were stained with Fixable Viability Dye eFluor™ 780. [file Image_6.tif]

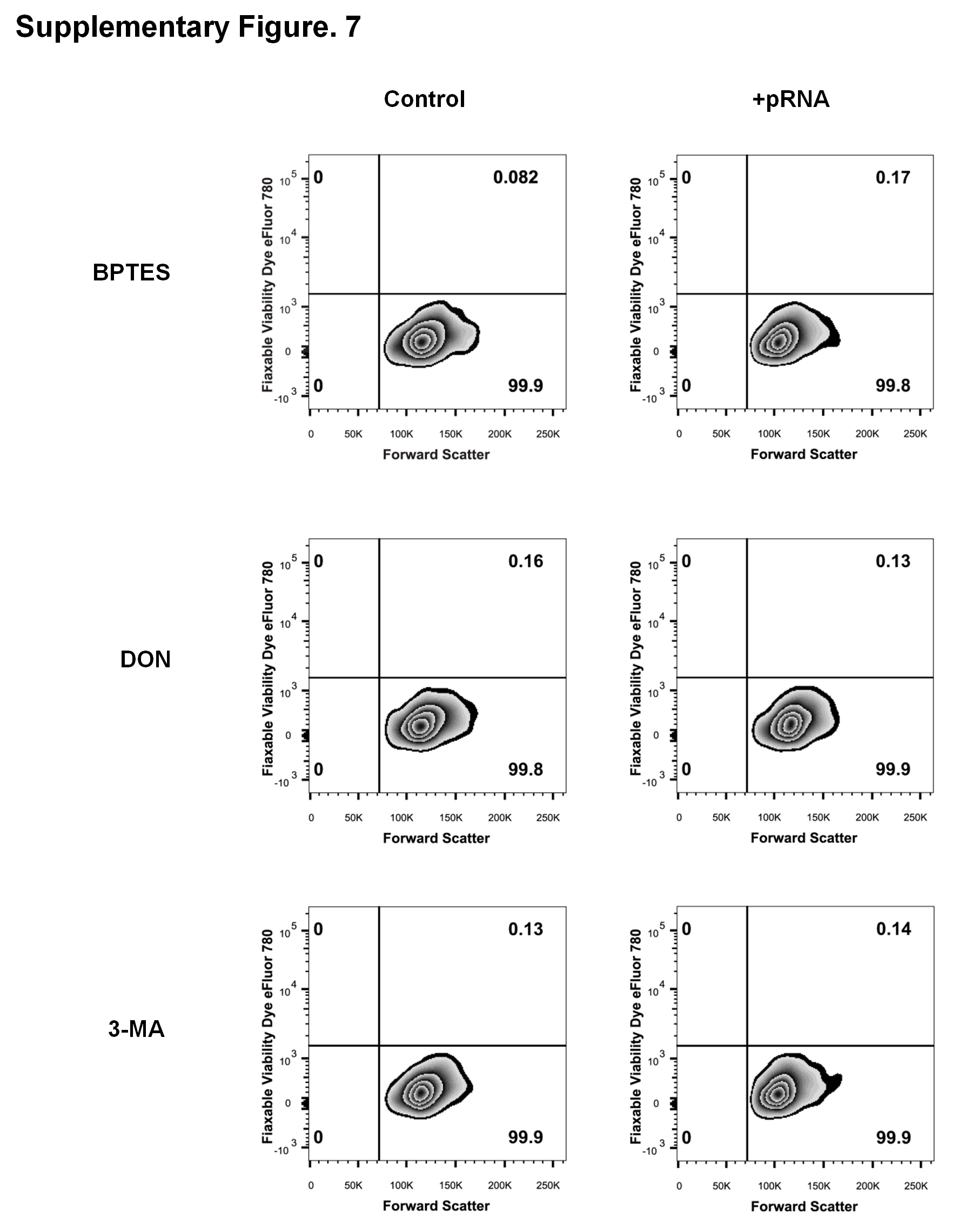

Supplement: Supplementary Figure 7 — PDC were stimulated with pRNA for 12 h in the presence or absence of 5 μM BPTES or 10 μM DON or 25 μM 3-MA. pDCs were stained with Fixable Viability Dye eFluor™ 780. [file Image_7.tif]

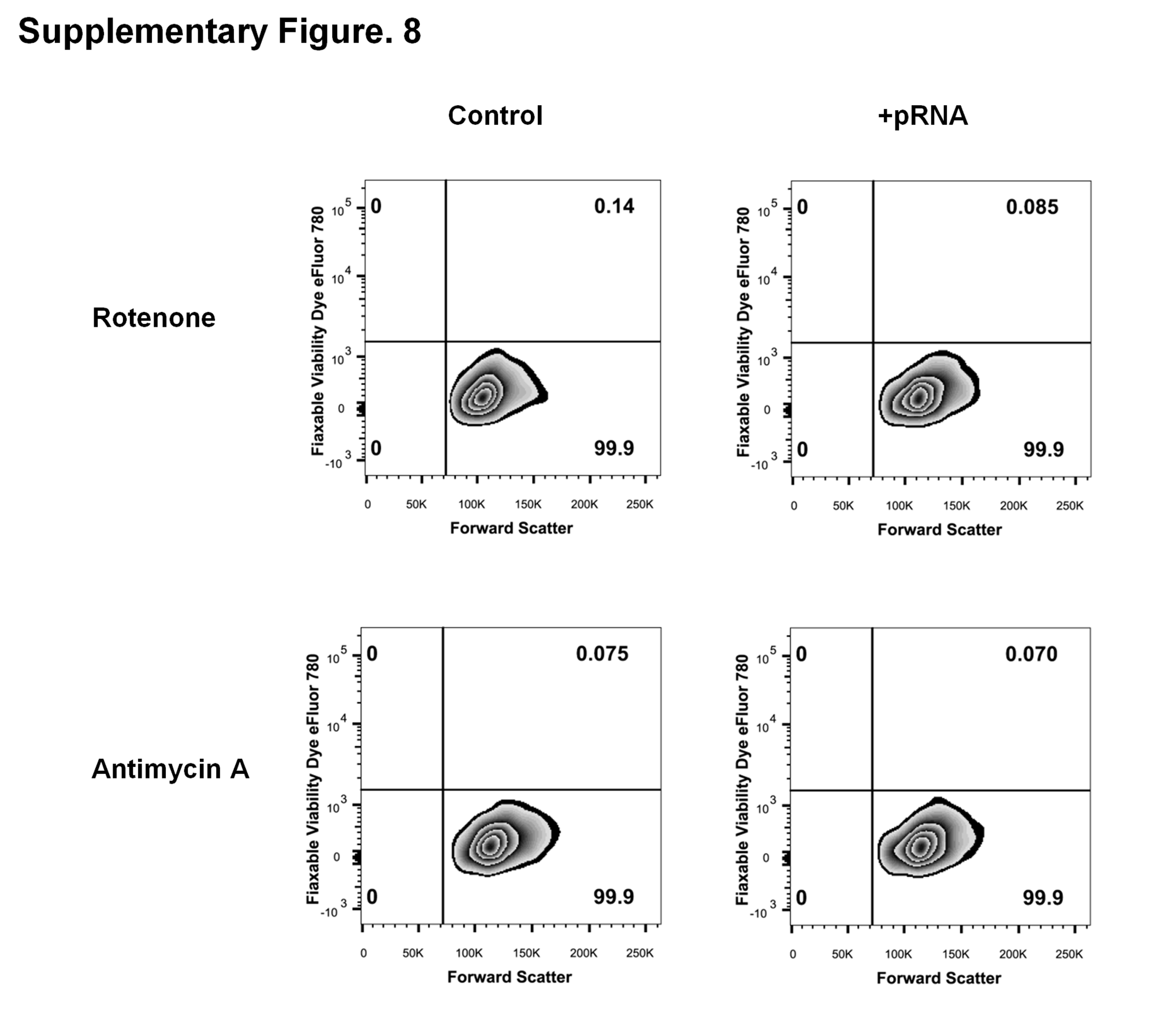

Supplement: Supplementary Figure 8 — PDC were stimulated with pRNA for 12 h in the presence or absence of 10 nM rotenone or 10 nM antimycin A. pDCs were stained with Fixable Viability Dye eFluor™ 780. [file Image_8.tif]
